# Nutritional management of children with acute kidney injury—clinical practice recommendations from the Pediatric Renal Nutrition Taskforce

**DOI:** 10.1007/s00467-023-05884-3

**Published:** 2023-03-20

**Authors:** Molly R. Wong Vega, Dana Cerminara, An Desloovere, Fabio Paglialonga, José Renken-Terhaerdt, Johan Vande Walle, Vanessa Shaw, Stella Stabouli, Caroline Elizabeth Anderson, Dieter Haffner, Christina L. Nelms, Nonnie Polderman, Leila Qizalbash, Jetta Tuokkola, Bradley A. Warady, Rukshana Shroff, Larry A. Greenbaum

**Affiliations:** 1 Texas Children’s Hospital, Houston, TX USA; 2 University Hospital Ghent, Ghent, Belgium; 3 Fondazione IRCCS Ca’ Granda Ospedale Maggiore Policlinico, Milan, Italy; 4 Wilhemina Children’s Hospital, University Medical Center Utrecht, Utrecht, The Netherlands; 5 University College London Great Ormond Street Hospital Institute of Child Health, London, UK; 6 1st Department of Pediatrics, Aristotle University, Hippokratio Hospital, Thessaloniki, Greece; 7 University Hospital Southampton NHS Foundation Trust, Southampton, UK; 8 Hannover Medical School, Children’s Hospital, Hannover, Germany; 9 Children’s Mercy Kansas City, Kansas City, MO USA; 10 British Columbia Children’s Hospital, Vancouver, Canada; 11 Great Northern Children’s Hospital, Newcastle Upon Tyne, UK; 12 New Children’s Hospital and Clinical Nutrition Unit, Internal Medicine and Rehabilitation, University of Helsinki and Helsinki University Hospital, Helsinki, Finland; 13 Emory University, Atlanta, GA USA; 14 Children’s Healthcare of Atlanta, Atlanta, GA USA

**Keywords:** Acute kidney injury, Nutrition, Continuous kidney replacement therapy, Pediatric Renal Nutrition Taskforce, Clinical practice recommendations

## Abstract

The nutritional management of children with acute kidney injury (AKI) is complex. The dynamic nature of AKI necessitates frequent nutritional assessments and adjustments in management. Dietitians providing medical nutrition therapies to this patient population must consider the interaction of medical treatments and AKI status to effectively support both the nutrition status of patients with AKI as well as limit adverse metabolic derangements associated with inappropriately prescribed nutrition support. The Pediatric Renal Nutrition Taskforce (PRNT), an international team of pediatric renal dietitians and pediatric nephrologists, has developed clinical practice recommendations (CPR) for the nutritional management of children with AKI. We address the need for intensive collaboration between dietitians and physicians so that nutritional management is optimized in line with AKI medical treatments. We focus on key challenges faced by dietitians regarding nutrition assessment. Furthermore, we address how nutrition support should be provided to children with AKI while taking into account the effect of various medical treatment modalities of AKI on nutritional needs. Given the poor quality of evidence available, a Delphi survey was conducted to seek consensus from international experts. Statements with a low grade or those that are opinion-based must be carefully considered and adapted to individual patient needs, based on the clinical judgment of the treating physician and dietitian. Research recommendations are provided. CPRs will be regularly audited and updated by the PRNT.

**Supplementary Information:**

The online version contains supplementary material available at 10.1007/s00467-023-05884-3.

## Introduction

Children with acute kidney injury (AKI) are at high risk for malnutrition and nutritional deterioration due to the high impact that electrolytes, minerals, protein, and fluid from nutritional sources have on the metabolic anomalies seen in AKI. As such, the nutritional assessment and nutritional management of AKI are as dynamic as its disease process. Appropriate nutritional therapy of AKI in children can limit metabolic and fluid derangements while continuing to provide optimal nutrition that supports healing. However, nutritional management in children with AKI lacks standardization, which is especially challenging for healthcare professionals given AKI’s phenotypic variation and the paucity of nutrition-based research.

The Pediatric Renal Nutrition Taskforce (PRNT), an international team of pediatric renal dietitians and pediatric nephrologists, has developed clinical practice recommendations (CPRs) addressing nutritional assessment and nutritional therapy in children with AKI. This CPR is based on an extensive review of the literature and, with the acceptance of there being limited evidence in the nutritional management of pediatric AKI, expert opinion employing adaptation of other areas of the literature (e.g., pediatric chronic kidney disease, pediatric critical care, adult acute kidney disease).

## Methods

Previous PRNT publications have described in detail the guideline development process, including working group composition and task distribution [1]. Abbreviations that are frequently used in the manuscript are listed in Table 1.

**Table 1:** List of abbreviations used in the manuscript with their full forms

Abbreviation	Full form
AKI	Acute kidney injury
PRNT	Pediatric Renal Nutrition Taskforce
CPR	Clinical practice recommendation
PICO	Patient group, intervention, comparator, outcome
KRT	Kidney replacement therapy
PICU	Pediatric intensive care unit
SDI	Suggested dietary intake
CKD2-5D	Chronic kidney disease, stages 2 through 5, including dialysis
ICU	Intensive care unit
CKRT	Continuous kidney replacement therapy
PYMS	Paediatric Yorkhill Malnutrition Score
STAMP	Screening tool for the assessment of malnutrition in paediatrics
PNST	Paediatric nutrition screening tool
PN	Parenteral nutrition
GI	Gastrointestinal
REE	Resting energy expenditure
MREE	Measured resting energy expenditure
IC	Indirect calorimetry
CHO	Carbohydrate
KDOQI	Kidney disease outcomes quality initiative
UL	Upper level

### Developing the PICO questions

Clinical practice recommendations provide precise actionable guidance on choosing between alternative approaches in specific clinical situations. We developed clinical questions to be addressed by each statement and framed them in a searchable format, with the specification of the patient group (P) to whom the statement would apply, the intervention (I) being considered, the comparator (C) (which may be “no action” or an alternative intervention), and the outcomes (O) affected by the intervention. Our PICO terms were as follows:

- Population: Infants, 37 or more weeks gestation, to children 18 years of age, with AKI
- Intervention: Nutritional support or assessment in children with AKI, with or without kidney replacement therapy (KRT)
- Comparator: Children without AKI or no comparator
- Outcomes: Optimization of nutrition to limit catabolism, limit metabolic complications, and maintenance or improvement, when necessary, of nutritional status

### Literature search

An electronic search using PubMed and an inclusive academic library search (including MEDLINE, Cochrane, and EMBASE databases) was performed using the search terms and strategy detailed in Supplementary Table 1. Limits were preset to only include manuscripts published in the English language between 1980 and August 2022. Given the paucity of studies in this field, all publications, including meta-analyses, prospective observational studies (irrespective of patient numbers), and retrospective observational studies and case series, have been included.

### Framing advice

The first PRNT-published CPR has outlined the development process and purpose of the recommendations [1]. Statements have been graded using the American Academy of Pediatrics grading matrix (Supplementary Table 2) and were submitted to a Delphi procedure, as previously described [1], to validate expert opinion.

## Clinical practice recommendations and rationale

Throughout this CPR, a critically ill child is defined as one requiring treatment in the pediatric intensive care unit (PICU) or comparable hospital unit (e.g., cardiac intensive care unit). Both critically ill and non-critically ill children may be receiving dialysis. AKI is defined based on laboratory and clinical criteria and was assumed to be based on an AKI guideline definition, which the PNRT recognizes may be modified over time [2]. Furthermore, given the evidence limitations, extrapolation and use of the suggested dietary intake (SDI) in children with chronic kidney disease stages 2 through 5, including dialysis (CKD2–5D) for a more pragmatic and international approach as described by Shaw and colleagues was used when referring to energy and protein CPRs [3].

1. Collaboration
  1.1 Ensure close collaboration between the healthcare professionals providing medical management and those providing the nutritional prescription for the optimal overall care of children with AKI. (level X; strong recommendation)

*Rationale:* Close collaboration between healthcare professionals is essential for the nutritional management of children with AKI in order to properly optimize nutrition provision and to determine the correct nutrition prescription and goals that align with the medical management and necessary treatment modalities. This is especially important in this patient population since major changes in clinical status or management occur frequently and may directly affect the nutritional prescription. Yet, especially in the intensive care unit (ICU) setting, there may be a variety of providers making medical decisions and other providers modifying the nutritional prescription. Medical decisions that may affect the nutritional prescription include stopping or starting dialysis, changing the dialysis prescription, and stopping or starting diuretics. Changes in clinical status may include volume overload or volume depletion, abnormalities in gut motility or absorption, and laboratory evidence or symptoms of uremia. Highly effective communication has the potential to improve patient safety [4]. The medical treatment of AKI is dynamic and nutritional therapy should reflect the dynamic nature of the medical treatment. Collaboration between medical and nutrition support teams spans the entirety of our recommendations and is thus a defining theme we will discuss throughout.

2. Nutritional assessment
  2.1 Utilize a validated pediatric nutrition risk screening tool for the assessment of nutritional risk within 48 h of AKI diagnosis. (level B, moderate recommendation)
  2.2 Refer any patient found to be at nutritional risk to a dietitian for nutritional assessment. (level B; moderate recommendation)
  2.3 Repeat nutritional assessments in accordance with the severity of nutritional risk, severity and duration of AKI, and changes in KRT. (level D; weak recommendation)

*Rationale:* The prevalence of malnutrition in hospitalized children ranges between 6 and 32%, depending on the country [5]. Children with the most severe AKI, requiring continuous kidney replacement therapy (CKRT), have reported malnutrition rates between 30 and 55% [6–9]. Fluid and electrolyte restrictions limit the selection of commonly consumed foods by children and impact nutritional status. Furthermore, it has been reported that critically ill children with AKI experience high rates of nutritional debt within the first 5 days of PICU admission [10]. In hospitalized children, malnutrition can increase adverse outcomes such as mortality, length of stay [11], readmission rates [12, 13], and infection rates [14], and it disproportionally affects children with severe medical conditions [11]. Due to the epidemiological shift of AKI in the last 20 years, there is likely to be a substantial overlap between children experiencing AKI and those having severe medical conditions such as multiorgan system failure, septicemia, respiratory failure, and congenital cardiac anomalies. A study on nutrition screening in hospitalized adults reported that high-risk patients identified via nutrition screening tools exhibited a higher incidence of AKI when compared to patients with normal nutritional status [15]. Therefore, we expect that children with AKI are at high risk for malnutrition and should have appropriate nutritional screening as detailed below.

Nutrition screening is an important first step that serves to notify a dietitian that a patient may be at nutritional risk and that further assessment of nutritional status is warranted. Only recently has there been the evidence to allow more informed decisions on the appropriate use of validated and reliable nutrition screening tools in hospitalized children. Four tools (Paediatric Yorkhill Malnutrition Score, PYMS; Screening Tool for the Assessment of Malnutrition in Paediatrics, STAMP; STRONGkids; and Paediatric Nutrition Screening Tool, PNST) have the most evidence to support their use with moderate validity [16–18]. However, it is important to note that none is specific to children experiencing AKI. Furthermore, the heavy reliance on anthropometric measurements of weight or proportionality of weight to height should be used with caution in children with AKI because of the potential for false positives or negatives in the case of patients with dehydration or fluid overload, respectively. Additionally, PYMS, STAMP, and STRONGkids include screening for high-risk diseases with nutritional impact and specify kidney disease, although not necessarily AKI. Yet, there is no reason not to utilize these screening tools in children with AKI.

Most groups recommend a 48 h screening period after admission to allow for timely nutrition evaluation and intervention if needed [18]. Timely intervention would allow nutrition teams to decrease the nutritional debt or limit the amount of time children with AKI are not meeting their optimal nutritional needs. This could also allow for the implementation of preventative strategies that support optimizing fluid balance and avoiding or minimizing electrolyte derangements. There is no consensus on the frequency of reassessment for hospitalized children other than via weekly re-screening of nutritional risk. However, given the dynamic nature of AKI and its treatment modalities, the reassessment of nutritional risk and efficacy of nutritional interventions should reflect the frequency with which the medical management of AKI changes. Furthermore, given the higher risk for malnutrition in younger children [11] and critically ill children [19], it is also expected that age and the presence of critical illness will also dictate the frequency at which children are reassessed for nutritional risk. Therefore, strong multidisciplinary collaboration is necessary to provide optimal nutritional management, which may require daily reassessment in some patients, especially those in the ICU.

2.4 Obtain accurate anthropometric measurements as soon as feasible and throughout hospitalization. (level A; strong recommendation) 
  2.4.1 Estimate euvolemic weight using accurate trended weight measurements in conjunction with other clinical assessment measures such as fluid balance, blood pressure, physical examination, and available biometric tools (e.g., bioelectrical impedance analysis, mid-upper arm circumference, non-invasive blood volume monitoring). (level D; weak recommendation)
  2.4.2 Measure height or recumbent length for children under 2 years of age. For those unable to stand for accurate measurement of height, use recumbent length or a surrogate missed measurement of height. (level A; strong recommendation)
  2.4.3 Measure head circumference for children up to 2 years of age or up to 3 years of age when appropriate centile charts are available. (level A; strong recommendation)
  2.4.4 Assess for muscle wasting by physical assessment and use of biometric tools where available. (level D; weak recommendation)

*Rationale*: Accurate evaluation of a hospitalized child’s nutritional status hinges on the appropriate evaluation of anthropometrics and body composition. As hospitalization and critical illness promote muscle protein breakdown in excess of muscle protein synthesis, children with AKI will experience negative alterations in fat mass, muscle mass, and functional capacity, thereby worsening morbidities and promoting long-term metabolic abnormalities in pediatric AKI survivors. Measurement of anthropometrics would ideally occur upon hospital admission, but are imperative once a child is diagnosed with AKI. It is highly likely that hypervolemia will mask muscle loss or true weight loss experienced by these patients. The most basic anthropometrics required for a full nutrition assessment to evaluate for malnutrition (acute or chronic) are weight and length/height [and head circumference for children less than 3 years of age] as per standard practice even outside of a diagnosis of AKI. Optimal nutritional management will attempt to limit or rectify malnutrition and malnutrition risk. Anthropometrics are a vital component for this evaluation. In an ideal setting, measurement of functional capacity and strength of a child with AKI to aid in the assessment of malnutrition would be performed. Unfortunately, anthropometrics are obtained at low frequency and accuracy in hospitalized children, worsening with critical illness [20, 21]. Children with AKI are most at risk for infrequent and inaccurate anthropometrics; the most frequent reasons reported for inability to obtain anthropometrics include hemodynamic instability and extracorporeal therapies. Furthermore, children with AKI frequently have fluid overload, which interferes with an evaluation of nutritional status when weight is used alone. Thus, the use of surrogate measures, including for height, may be necessary, as has been recommended previously in children with CKD [22]. Individualized nutritional prescription relies on monitoring changes in nutritional status, and if changes are not accurately quantified, then children may not receive optimal nutritional prescriptions. Thus, evaluation of a child’s nutritional status and body composition (namely muscle mass and volume status) should be performed using a variety of metrics.

Assessment parameters such as net fluid balances, bioelectrical impedance analysis (BIA), ultrasound, musculoskeletal physical assessment, non-invasive blood monitoring in acute hemodialysis (HD), and mid-upper arm circumference (MUAC) may provide alternative means to support the assessment of nutritional status in the absence of accurate anthropometric measurements. Table 2 provides examples of a variety of body composition (muscle, fat, and water) techniques that may be available for comprehensive nutritional status evaluation in the setting of shifting fluid status to support individualized therapeutic nutrition interventions; these are consistent with CKD assessment recommendations [22]. A physical assessment of muscle wasting can predict the longer length of stays independently of anthropometrics or other nutritionally-related risk factors of malnutrition in hospitalized children [39]. MUAC has been revitalized in the pediatric malnutrition literature and may hold promise to aid the assessment of malnutrition [32, 40, 41]; it is a quick and easy anthropometric tool, especially when weight and height measurements are not available in immobilized patients [42]. Interestingly, MUAC has been reported to be less affected by fluid status changes than weight, especially in the setting of hypovolemia [43, 44]. Volume status can be estimated via physical assessment and net fluid balance. However, for institutions with more resource availability, the utilization of biometric tools such as ultrasound or BIA may improve the assessment of volume status and muscle mass changes. BIA has been utilized to evaluate a variety of clinical parameters including both fluid and nutritional status in critically ill children [19, 45]. Ultrasound has also been utilized in a variety of ways. Some studies have assessed fluid status through point-of-care lung ultrasound testing [46] or inferior vena cava diameter [31], while others have investigated muscle thickness changes in critically ill children [47, 48] to monitor nutritional status in the absence of other anthropometrics. Adaptations and advances in technology will be necessary to allow for improvements in assessment methods of nutritional status and the efficacy of individualized nutritional therapies.

**Table 2:** Multifocal body composition and body compartment assessment techniques available for hospitalized children

Method	Body Compartment	Advantage	Limitation
BIA [23, 24]	Fat mass, fat free mass estimated from total body water	Quick, portable, inexpensive, safe, reproducible, non-invasive, does not require highly trained personnel, Increasing evidence in utilization of fluid status assessment [25, 26]	Sensitive to hydration, individual level prediction errors, limitations to prediction equations (e.g., sex, age, ethnicity), limited standardization of reportable measures
CT [23, 27]	Adipose tissue, skeletal muscle, bone, organs	Accurate, L3 cross sectional area good correlation with total body muscle volume	Radiation exposure, costly, requires expertise, body size limitation, availability of site and disease state
MRI [23, 27]	Adipose tissue, skeletal muscle, visceral organs	Accurate	Costly, requires expertise, not portable
B-Mode ultrasound [23, 27–29]	Localized muscle thickness and fatty infiltration Localized skin thickness – multisite equations for FM estimations Fluid overload – lung scoring [30], inferior vena cava diameter [31]	Portable, safe, minimal training, reproducible, beneficial for abdominal adiposity discrimination between subcutaneous and visceral May differentiate volume-dependent hypertension from volume-independent hypertension in dialysis	Muscle measurement more prone to error due to compressibility, site selection, transducer position and hydration, secondary processing required for fatty infiltration of muscle, 2 dimensional Fluid assessment not reliable, not correlated with extracellular fluid volume
Anthropometry	Weight/Length – fat estimation BMI – fat estimation SFT – fat estimation Circumferences: MUAC – total body muscle estimation [32, 33] Leg – site specific muscle estimation Arm – site specific muscle estimation	Quick, portable, easy, inexpensive, non-invasive, safe SFT performs well in CKD MUAC with good correlation to BMI z scores ≤ –2 [32]	Insensitive, altered by hydration, best used serially, dependent on accurate weight and height measurements Difficulty attaining accurate weight and height measures in ICU SFT with operator variability, performs poorly in dialysis [34]
Physical assessment [14, 35, 36]	Subjective surrogate measures for change in fat, muscle, and edema	Reliable, clinically validated	Requires additional measures to confirm assessment
% Fluid overload [37]	Relative TBW %FO = ((Fluid in – Fluid out)/ ICU admission wt) × 100 Weight modified (CKRT) %FO = ((CKRT initiation wt – ICU Admission wt)/ ICU admission wt) × 100 %FO = ((CKRT initiation wt –hospital wt)/ hospital admission wt) × 100	Quick, easy to perform, validated	Reliant on precise and accurate I/O measurement and calculations, does not account for insensible losses, requires accurate weight measurements, fluid accumulation not site specific
Non-Invasive Blood Volume Monitoring [38]	relative blood volume; hematocrit dilution changes during hemodialysis	Validated method to achieving euvolemic weights while limiting dialysis-associated morbidities	Specific to hemodialysis, requires specialized equipment

*BIA*, bioelectrical impedance analysis; *CT*, computed tomography; *MRI*, magnetic resonance imaging; *L3*, lumbar vertebra 3; *BMI*, body mass index; *SFT*, skin fold thickness; *CKD*, chronic kidney disease; *ICU*, intensive care unit; *MUAC*, mid-upper arm circumference; *FO*, fluid overload; *CKRT*, continuous kidney replacement therapy; *wt*, weight; *I/O*, ins and outs; *TBW*, total body water

Adapted from multiple sources within table

The standard nutritional practice recommends nutritional reassessments of hospitalized children weekly, at minimum. However, there are no definitive recommendations on the frequency of anthropometric evaluation by nutrition support societies [49]. Anthropometric and body composition evaluation are key components to aid in the monitoring and evaluation of the appropriateness of nutritional therapies. Therefore, the frequency of anthropometric measures should continue to reflect the dynamic changes in AKI, its corresponding medical management, and the acuity and age of the child. A weight measurement of smaller children and infants should occur more frequently. A weight measurement of children where fluid overload has masked euvolemic weights may be considered more frequently to refine nutrition prescriptions. Measures reflecting anthropometrics or body composition changes that may change more slowly may be done at lower frequencies.

3. Oral and enteral feeding
  3.1 Oral feeding, including breastfeeding, is the preferred method of providing nutrition. (level X; strong recommendation)
  3.2 In critically ill children, consider early initiation (within 48 h of admission) of supplemental or exclusive enteral tube feeding when oral feeding does not meet nutritional requirements, especially when nutritional intake is likely to remain suboptimal. (level C; weak recommendation)
  3.3 Use whole protein (polymeric) formulas unless otherwise indicated, such as in the case of gastrointestinal dysfunction. (level C; weak recommendation)
  3.4 Consider the use of protein and energy-dense formulas to achieve nutritional goals within the limits of the fluid allowance and gastrointestinal tolerance; adjust formula density gradually to maximize tolerance. (level C; moderate recommendation).
4. Parenteral nutrition
  4.1 For children with malnutrition or risk for nutrition deterioration, when oral or enteral nutrition cannot provide all nutritional requirements, initiate supplemental parenteral nutrition. (PN) (level X, moderate recommendation)
  4.2 For children without malnutrition or risk for nutrition deterioration, when oral or enteral nutrition cannot provide all nutritional requirements, PN may be withheld for up to 1 week provided micronutrients are delivered. (level B; moderate recommendation)
  4.3 For all children, regardless of nutritional status, receiving KRT that causes significant nutrient losses, initiation of PN before 1 week should be considered when oral or enteral nutrition cannot provide all nutritional requirements. (level D; weak recommendation)
    4.3.1 Pay careful attention when transitioning from PN to enteral feeding in children receiving CKRT to ensure the provision of optimal nutrition within the fluid allowance. (level C; moderate recommendation)

*Rationale*: Feeding through the gastrointestinal (GI) tract remains important to decrease morbidity in hospitalized children. Critically ill children who receive enteral feeding early in their management, especially feeding associated with higher protein intakes (> 60% of their prescribed goal during their PICU stay), exhibit lower mortality rates [50].

Breastmilk contains the optimal energy and nutrient composition to promote health and age-appropriate growth in infants and toddlers [51, 52], and the PRNT supports breast feeding or giving expressed breastmilk whenever possible [3]. The use of energy-dense formula is a key nutrition intervention, often applied in children with AKI, especially when fluid restriction is necessary for optimal medical management. Energy-dense oral nutrition supplements should be offered to children not meeting nutritional needs with an oral diet alone. In a randomized controlled trial of children with faltering growth, energy-dense oral nutrition supplementation has been shown to result in significantly higher energy intake compared to lower-density oral nutrition supplements [53]. Furthermore, the use of energy-dense formula in critically ill adults has been shown to meet nutritional needs sooner due to the ability to achieve goal energy needs in less time, allowing for meeting nutritional goals despite procedural disruptions [54]. The introduction of energy-dense enteral tube feeding should occur gradually to promote GI tolerance [3, 55]. There is insufficient evidence to support the selection of partially hydrolyzed or extensively hydrolyzed protein formulas over whole protein (polymeric) formulas unless the latter are contraindicated or poorly tolerated [49]. However, there are also reports that children with AKI exhibit higher rates of GI intolerance [56]. Additionally, patients with sepsis-related AKI may frequently experience GI intolerance [57].

When oral feeding fails to meet nutritional requirements and enteral tube feeding is necessary, there is not enough evidence to recommend the optimal tube placement site, but the gastric route is preferred for patients in the PICU as compared to the post-pyloric route [3, 55]. There is no evidence to support the use of continuous over intermittent feeding in critically ill children [49]. Despite the reported benefits of enteral feeding, it may be a barrier to the provision of adequate protein in children with high protein: calorie ratio needs, such as those receiving CKRT [58]. However, enteral feeding, even at trophic levels, promotes gut barrier function, thereby limiting potential infectious complications [59–61].

In agreement with current evidence on initiation of PN when sufficient nutrition via the enteral route cannot be achieved, in the absence of malnutrition or KRT, PN initiation may be delayed up to 1 week, provided micronutrients are delivered prior to that time. Current evidence indicates that early (< 24 h) PN initiation is associated with worse outcomes (longer length of stay and increased infection rates) [59]. In early PN initiation, amino acids (ranging from 0.75 to 1.15 g/kg per day) were associated with increased infections and longer dependency on mechanical ventilation [60]. The evidence supporting the avoidance of early PN initiation is based on a single randomized clinical trial in critically ill children where PN was initiated if enteral goals did not provide more than 80% of their caloric target. Admittedly, this is a relatively high goal within the first week of admission to a PICU given the metabolic changes that occur in such patients. Evidence supporting a more pragmatic timeframe to achieve therapeutic nutrition goals is limited to the critical care literature in children, where achieving approximately 60% of energy and protein targets within 7 days of a PICU admission via enteral or enteral with supplemental PN is associated with lower mortality, but not ventilator-free days or infections [62]. It is hypothesized that withholding early PN support during the acute phase of critical illness maintains autophagy, an essential survival mechanism, which decreases the risk of organ failure and cell death; autophagy also has an important role in innate immunity [63]. In children with AKI, where there are higher rates of malnutrition, GI disturbance, and increased dialysis nutrient-related losses, PN may need to be considered earlier than 1 week given their higher overall risk for nutrition-related disturbances.

5. Energy requirements
  5.1 non-critically ill children, the initial prescription for energy intake should approximate the SDI based on euvolemic weight, not measured weight. (level B; moderate recommendation)
  5.2 In the acute phase of critical illness, energy requirements should not exceed the resting energy expenditure (REE). (level C; weak recommendation)
  5.3 In the stable phase and recovery phase of critical illness, the energy prescription must account for energy debt, physical activity, rehabilitation, and growth. (level X; moderate recommendation)
  5.4 Modify the energy prescription to account for dialysis-related net gain or loss of energy. (level C; weak recommendation)
  5.5 In critical illness, consider an increased percentage of energy intake from fat to reflect changes in beta-oxidation when parenterally fed. (level C; weak recommendation)

*Rationale*: For non-critically ill children with AKI, the SDI for energy can guide the initial prescription of dietary intake [3]. During critical illness, the body reacts to physiological stress in three phases characterized by different metabolic responses [64]. In the acute phase, where vital organ support is required, muscle protein synthesis and REE decrease. The body reacts with an inflammatory cascade aiming to survive the critical illness by supplying blood, energy, and substrates to the injured site [65]. The metabolic response is catabolism (muscle protein breakdown and lipolysis) for substrate delivery to the vital tissues. Hyperglycaemia occurs due to increased gluconeogenesis and peripheral insulin resistance [66]. Catabolism and muscle protein breakdown is not reversed with increased provision of nutrients during this phase despite previous ideas to the contrary [67]. In the stable phase, there is maintenance or weaning of vital organ support, but not all aspects of the stress response are resolved [64]. There is still protein wasting despite the small increase of anabolic hormones such as growth hormone and insulin-like growth factor-1. Muscle wasting is exacerbated by immobilization and medication [68]. During the recovery phase, there is no longer a need for vital organ support, the child is mobilized, and the stress response is resolved. Hormone levels normalize and the body shifts from catabolism to anabolism (positive nitrogen balance, tissue repair, and catch-up growth) [64, 69]. During the stable and recovery phases, the aim of nutritional support is to restore lean body mass [64].

Little is known about the energy requirements of critically ill children with AKI. Based on observational cohort studies in critically ill children (not AKI exclusively), measured REE (MREE) by indirect calorimetry (IC) is the best diagnostic technique to determine the energy requirements during the acute phase. However, IC is not widely available nor feasible, so the Schofield weight-height or Schofield weight (see Table 3) or the World Health Organization equations without the addition of stress factors may be used to determine EE during the acute phase of illness [49, 55]. The Harris–Benedict equations and recommended dietary allowances should not be used to determine the energy requirement of critically ill children. No correlation has been found between MREE and nutritional status, initial diagnosis, or severity of the acute illness [49, 55, 72–75]. We suggest that IC be used to determine the EE in critically ill children with AKI. In the absence of IC, either the Schofield or WHO equations (without stress factors) may also be used [76]. REE can be increased when the child has a body temperature above 38 °C, or decreased when the child is deeply sedated. Energy intake should be increased gradually above the REE to ensure full recovery and catch-up during the stable and recovery phases. Furthermore, based on the prior discussion regarding avoidance of early PN initiation and the research available on appropriate timing to achieve target energy goals, it is likely that achieving greater than the REE would be difficult when following feeding modality guidance in critically ill children. To avoid over and underfeeding the critically ill child who is on CKRT, it may be important to account for the energy (calories) from citrate (3 kcal/g), lactate (3.62 kcal/g) and glucose from dialysis, hemofiltration or anticoagulation solutions [76]. The net caloric gain depends on the type and rate of fluids used as well as the CKRT dose [77]. Furthermore, acute peritoneal dialysis may also provide calories from glucose-containing dialysis solution; however, there are limited studies that address this issue [78, 79], and maintenance peritoneal dialysis nutrition practice is to avoid this calculation and adjust based on growth trends. In total, serial anthropometric evaluation works in concert to aid the assessment of energetic adequacy.

**Table 3:** Schofield [70] and WHO [71] equations for calculating REE

	Age	Boys	Girls
WHO	0–3 y	60.9 × (w kg) − 54	61.0 × (w kg) − 51
	3–10 y	22.7 × (w kg) − 495	22.5 × (w kg) + 486
	10–18 y	22.7 × (w kg) + 651	12.2 × (w kg) + 746
Schofield w	0–3 y	59.5 × (w kg) − 30	58.3 × (w kg) − 31
	3–10 y	22.7 × (w kg) + 505	20.3 × (w kg) + 486
	10–18 y	17.7 × (w kg) + 658	13.4 × (w kg) + 692
Schofield w/ l	0–3 y	0.167 × (w kg) + 1517.4 × (l m) − 616.6	16.252 × (w kg) + 1023.3 × (l m) − 413.5
	3–10 y	19.6 × (w kg) + 130.3 × (l m) + 414.9	16.25 × (w kg) + 161.8 × (l m) + 371.2
	10–18 y	16.97 × (w kg) + 137.2 × (l m) + 515.5	8.365 × (w kg) + 465 × (l m) + 200.0

*WHO*, World Health Organization; *REE*, resting energy expenditure; *w*, weight; *l*, length

The optimal energy substrate in critically ill children with AKI is also not well known. However, in a cross-sectional study with 33 critically ill children (non-AKI) receiving PN and mechanical ventilation, fat was used preferentially for oxidation, and carbohydrate (CHO) was utilized poorly [80]. A higher CHO intake promotes lipogenesis and decreases lipid oxidation. In a prospective multicenter study on 42 adult patients with AKI receiving enteral and/or PN, CHO oxidation was significantly lower than both prescribed and administered CHO. This study showed that adults with AKI used much less CHO than expected, while oxidizing much more lipid [81]. An increased insulin resistance leads to hyperglycemia and increases lipolysis leading to increased beta-oxidation, indicating a potential shift in substrate utilization because of the inflammatory milieu from critical illness and AKI. The precise composition of CHO and lipid substrate delivery could prevent worsening metabolic derangements, and thus, in children with AKI, increased lipid over CHO administration may be more appropriate.

6. Protein requirements
  6.1 For non-critically ill children, the initial prescription for protein intake should approximate the SDI based on euvolemic weight, not measured weight. (level B; moderate recommendation)
  6.2 critically ill children, consider the potential need for increased protein intake above the SDI to limit negative protein balance. (level B; moderate recommendation)
  6.3 In children with very elevated blood urea nitrogen levels, especially if progressively worsening, first ensure adequate energy intake; then, consider a temporary lowering of protein intake towards the lower end of the SDI. (level C; moderate recommendation)
    6.3.1 Do not persistently compromise protein intake to lower urea nitrogen levels or postpone KRT initiation. (level X; strong recommendation)
  6.4 For all children receiving dialysis, protein prescription needs to be further increased to account for dialysis losses, which are highest in CKRT. (level C; moderate recommendation)

*Rationale*: Baseline protein need is higher in children than in adults given the need for growth. Updated consensus recommendations support the use of a pragmatic and international approach to appropriate protein provision in pediatric CKD2–5D utilizing the SDI [3]. In the setting of limited evidence, for non-critically ill children, the SDI guide protein recommendations. Catabolism and negative nitrogen balances are frequently reported in children [7, 82, 83] and adults [84–87] with AKI. In the setting of AKI, the inflammatory milieu and changes to the ubiquitin–proteasome system pathway can increase muscle protein breakdown, which leads to higher urea nitrogen production and thus negative nitrogen balances [88]. Adequate protein provision aims to attenuate muscle protein breakdown, support tissue repair, and facilitate rehabilitation leading to better functional outcomes of survivors. It is important to consider how healthcare professionals aim to achieve optimal protein intake in children with AKI. Although enteral protein provision has been associated with lower ICU mortality [50, 62], enteral formulas may not contain the optimal protein concentration for critically ill children receiving CKRT; hence, they may require additional protein modular supplementation or even supplemental PN to achieve protein goals [58].

No data exists on the appropriate protein dosing for children receiving conservative medical management for AKI. However, restriction of protein which would cause worsening malnutrition to delay dialysis initiation is not recommended by pediatric nephrology healthcare professionals [3]. There is no data to support that protein provision inhibits AKI recovery. In the CKD literature, a randomized controlled trial comparing protein intakes showed that lower length and growth velocity were observed in children with lower protein intakes [89]. Moreover, it is well established that lower height attainment is associated with greater mortality in children with CKD [90]. We acknowledge that data from CKD are likely not fully comparable for those children with AKI. However, in AKI contributing factors to elevated blood urea nitrogen levels in the setting of catabolism, especially beyond the acute phase of critical illness, should be evaluated closely as muscle protein breakdown also acts as a source of urea generation. Any decreases in protein intake for extended periods of time need to be carefully evaluated for their utility and patient-centered goals of care.

Protein needs in acute dialytic therapies in AKI treatment are often extrapolated from chronic dialytic therapy. Studies of children receiving chronic peritoneal dialysis have shown protein losses may range from 0.1 to 0.28 g/kg/day, with the highest losses seen in smaller children [3, 91], while achievement of positive nitrogen balances required up to approximately 144% of the recommended daily allowance [92] in the setting of steady-state energy and protein metabolism. Through extrapolation of adult maintenance (3 times/week) HD studies, the Kidney Disease Outcomes Quality Initiative (KDOQI) workgroup recommended 0.1 g/kg/d to account for dialysis-related protein losses in children [93]. The PRNT recommendations for energy and protein for children with CKD2–5D updated these recommendations through the utilization of the SDI ranges when accounting for these losses [3].

Most of the data available regarding the nutritional management of patients with AKI addresses amino acid losses experienced in CKRT. These studies consistently reported losses equivalent to approximately 10–20% of amino acids provided via nutrition support [7, 82, 94]. Studies of children receiving CKRT demonstrate negative nitrogen (protein) balance. For example, in a study where 120–130% of MREE and 2 g protein/kg/day were provided, negative protein balances persisted [82]. Additionally, concentrations of serum amino acids appear to stabilize 5 days following the initiation of CKRT, save glutamic acid [94, 95]. Our knowledge of protein losses experienced in CKRT only includes the initial 5 days after CKRT initiation. Protein losses from KRT should be accounted for to prevent negative protein balances.

7. Micronutrient needs (vitamins, trace elements, and carnitine)
  7.1 In children who are conservatively managed, provide the recommended requirements of vitamins and trace elements for healthy children, with supplementation in the case of insufficient intake. (level D; weak recommendation)
  7.2 In children requiring dialysis, consider providing additional supplemental water-soluble vitamins, selenium, copper, zinc, and carnitine; either enterally or parenterally. (level D; weak recommendation)
  7.3 Avoid supplemental vitamin A in all children with AKI. (level B; strong recommendation)
  7.4 Evaluate for clinical signs and symptoms of deficiency or excess of vitamins, trace elements, and carnitine. (level C; weak recommendation)
  7.5 Do not routinely measure serum concentrations of vitamins, trace elements, and carnitine unless there are clinical signs or symptoms of deficiency/toxicity, or when the child is receiving treatment for deficiency or has known toxic concentrations. (level D; weak recommendation)

*Rationale:*

### Vitamins

Critically ill children are prone to vitamin deficiencies due to a hypermetabolic state, decreased intestinal absorption, insufficient intake, increased excretion, medication-related effects, and underlying metabolic disorders. Patients on dialysis are also at risk of deficiency due to the loss of water-soluble vitamins and trace elements in the dialysate [96], although there is limited data on their clearance by individual dialysis modality. There is also limited data on the assessment of vitamin B status in critically ill pediatric patients [97]. Based on one study, children on CKRT are at risk for decreased folate and thiamine levels [96].

The current level of evidence is too low to make recommendations for the routine measurement of vitamin and trace element concentrations in patients with AKI. Laboratory evaluation of most micronutrients should be used with caution. Inflammation can affect many serum micronutrient levels due to movement between plasma and the intracellular space as well as decreased sequestration and increased exposure to reactive oxygen species [98]. However, the presence of symptoms of nutrient deficiency or excess is important to identify, especially in children with underlying CKD who may have suboptimal nutrition or additional risk factors for pre-existing nutritional deficiencies [96]. In a retrospective review of 47 children on maintenance dialysis who received daily supplementation with pyridoxine and a commercial water-soluble vitamin preparation, the concentrations of several trace elements and vitamins were outside the reference range, with both deficiency and excesses noted [99]. Similar data in children with AKI is not available. Moreover, as may be expected in the clinical setting of AKI, it is not known if an acute and transient deficiency of particular vitamins and trace elements can lead to adverse outcomes. The clinical signs of deficiencies or excess of vitamins and trace elements are described in Table 4. If clinical symptoms of micronutrient deficiency or excess are noted, laboratory assessment is suggested to confirm clinical findings and intervene appropriately. Repletion dosing used in the general pediatric population is reasonable, though monitoring to document successful correction may be appropriate (Supplementary Table 3).

**Table 4:** Overview of vitamins and trace minerals at risk for deficiency or excess in the pediatric dialysis population

Nutrient	Symptoms of deficiency	Symptoms of excess	Dietary sources	Diagnostic tests
Vitamin A	Night blindness, xerophthalmia, keratomalacia, poor bone growth, impaired resistance to infection, follicular hyperkeratosis	Hyperostosis, hepatomegaly, hepatic fibrosis, alopecia, increased cerebrospinal, fluid pressure [100], hypercalcemia [101]	Fortified milk, liver, egg, cheese, yellow fruits and vegetables (carotenoid precursors)	Plasma retinol [97]
Vitamin E	Hemolytic anemia in premature infants; fat malabsorption causes deficiency; hyporeflexia and spinocerebellar and retinal degeneration	Bleeding, impaired leukocyte function	Sardines, green and leafy vegetables, vegetable oil, wheat germ, whole grains, butter, liver, egg yolk	Plasma alpha-tocopherol [97]
Thiamine (B1)	“Wernicke encephalopathy”- peripheral neuropathy, ophthalmoplegia, nystagmus, ataxia, edema	Unknown	Dairy, nuts, legumes, fruits, egg, unrefined grains, pork, vegetables	Thiamine pyrophosphate level, whole blood/red blood cell transketolase activation test
Pyridoxine (B6)	Facial seborrhea, glossitis, angular stomatitis, cheilosis, mental status changes	Neuropathy, photosensitivity	Fortified cereals	Pyridoxal 5’-phosphate 4-pyridoxic acid
Folate (B9)	Abdominal cramps, nausea, diarrhea, irritability, poor sleep, seizures, megaloblastic anemia	Masking of B12 deficiency symptoms in patients with pernicious anemia not receiving cyanocobalamin (B12)	Green vegetables, liver, grains, some fruits	Acute deficiency – low serum folate; chronic deficiency – low red blood cell folate
Vitamin C (ascorbic acid)	“Scurvy”- osmotic diarrhea, gingival bleeding, perifollicular hemorrhage, long bone changes (arthropathy)	Massive doses predispose to kidney stones; nausea, abdominal pain; rebound scurvy when massive doses stopped	Citrus fruits	White blood cell ascorbate concentration, radiographic evidence of long bone widening
Zinc	Clinical phenotype of”Acrodermatitis enteropathica” – anorexia, hypogeusia, growth retardation, diffuse skin lesions with impaired wound healing	Few toxic effects; may aggravate marginal copper deficiency	Meat, dairy, legumes, whole grains, oysters	Blood zinc level
Selenium	“Keshan disease”- progressive cardiomyopathy, cardiovascular disease with thyroid disease, also with myositis	Irritation of mucous membranes, pallor irritability, indigestion	Meats, seafood, nuts	Blood selenium level
Copper	Sideroblastic anemia, retarded growth, osteoporosis, neutropenia, decreased pigmentation	Few toxic effects; Wilson disease, liver dysfunction	Shellfish, meat, legumes, nuts, cheese	Serum copper, ceruloplasmin, superoxide dismutase activity
Chromium [102]	Glucose intolerance, weight loss and a metabolic encephalopathy-like confusion state	Genotoxic and carcinogenic	Meat and meat products, oils and fats, breads and cereals, fish, pulses and spices	Serum, plasma, urine chromium levels
Manganese [103]	Fleeting dermatitis, miliaria crystallina	Neurological disorder	Nuts, chocolate, cereal-based products, crustaceans and molluscs, pulses, fruits and fruit products	None
Carnitine	Hypoglycemia, dyslipidemia, muscle weakness, rhabdomyolysis, cardiomyopathy, arrhythmia, sudden death	Diarrhea	Meat, fish, dairy	Plasma, urine, skeletal muscle

Table adapted from multiple sources [96, 102–106]

There is a paucity of research and high-quality evidence to guide micronutrient supplementation in AKI. The latter has led to large variations in practice around the world [99]. Requirements in children with AKI are compared against the recommended requirements for healthy children; however, caution should be exercised to avoid exceeding the upper levels (UL) when the intake of diet and supplement is combined. Children receiving the majority or all their energy requirements from adult renal formulas generally meet 100% of the recommended requirements for vitamins and trace elements and may not require vitamin supplementation [104]. Care should be taken not to exceed 100% of the recommended requirements for vitamin and trace element intake due to the potential for toxicity, particularly in the oligo-anuric dialysis patient [96]. Micronutrient losses may be cumulative, and the dietitian should keep the duration of AKI and specific KRT modality in mind when assessing micronutrient need.

Supplementing vitamin C in critically ill adults has a sound pathophysiological rationale and a positive safety profile. Patients on KRT likely need doses similar to those of critically ill patients not receiving KRT. Intravenous vitamin C may be necessary to achieve normal plasma concentrations during KRT. However, data on dose adjustment of vitamin C during intermittent or chronic KRT are sparse, and more pharmacokinetic and dose–response studies are required [107].

Supplementation doses should be individualized based on individual patient needs, risk profiles, and dialysis losses [105]. Assessment of serum vitamin B12 should be considered if folate supplementation is administered. High folic acid intake may mask signs of pernicious anemia and silent progression of neurologic disease; thus, both folate and vitamin B12 levels should be monitored if folate is being supplemented [105]. Monitoring of these levels may be considered at 2-week intervals if supplementation is initiated, as levels may normalize after 2 weeks of B12 supplementation.

The kidneys play an important role in the metabolism and excretion of vitamin A. Patients with impaired kidney function have high circulating levels of retinol, possibly due to a combination of decreased glomerular filtration of the retinol–retinol-binding protein complex, reduced conversion of retinol to retinoic acid, and an accumulation of retinol-binding protein [100]. Increased levels of retinol are reported in infants [108] and children with CKD and on maintenance dialysis despite lack of supplementation and are correlated with hypercalcemia [101]. Retinoic acid has been shown to reduce inflammation and fibrosis in experimental models of kidney injury; however, hypervitaminosis A has also been reported in AKI [109].

### Trace elements

Trace elements play a key antioxidant role in critical illness. Altered concentrations of trace elements during AKI are well described and are due to variable protein binding, redistribution from blood to tissues, acute losses, and removal by CKRT [110–112]. In the few studies that assessed effluent losses of trace elements in children with AKI, there was significant variation among studies, depending, in part, on the dialysis modality and its duration [94, 113, 114]. Trace element removal by CKRT, including zinc, copper, chromium, and selenium, was reported to be lower than the supplemental amounts received through PN, thus causing no deficiencies. However, two pediatric studies reported manganese excess but without evident clinical symptoms in the affected children [94, 113]. In the only prospective pediatric study, selenium balance was negative on day 2 and day 5 after initiation of CKRT [94]. Lower concentrations of selenium have been independently associated with an increased risk of death and hospitalization [115]. A prospective study in adults has shown that all trace elements studied were below the reference range throughout the 6-day study period, both in CKRT and non-CKRT patients [116]. Nevertheless, no study has provided high-quality evidence on the effect of supplementation, deficiencies, or excess of trace elements on the clinical outcome of critically ill pediatric or adult patients [86, 114, 117]. Repletion dosing as suggested in the general pediatric population is reasonable, though monitoring to document successful correction may be appropriate (Supplementary Table 3).

### Carnitine

The combination of a low protein intake together with the removal of carnitine by CKRT can lead to the depletion of levocarnitine (L-carnitine), which facilitates the transport of fatty acids across the inner mitochondrial membrane and is thus a critical co-factor for normal energy production in cardiac and skeletal muscle. Dialysis-related carnitine deficiency is common among children and adults on chronic HD due to the efficient removal of carnitine during each HD treatment, with an inverse relationship between muscle carnitine and duration on dialysis [118, 119]. Within a single dialysis session, clearance is 30 times greater than would be expected in a healthy individual. HD results in an abnormal acylcarnitine:free carnitine ratio [120]. In a pediatric cohort of 42 patients on CKRT, carnitine was even more rapidly depleted by CKRT compared with chronic HD, with losses approximating 80% of intake. Carnitine deficiency was associated with a longer duration of stay and increased mortality [106]. In a small pediatric study, CKRT patients who did not receive carnitine supplementation were carnitine deficient after 1 week on CKRT, while supplementation with intravenous carnitine was associated with repletion of plasma carnitine and improvement in myocardial strain [121]. Data are less conclusive in adult critically ill patients with AKI starting CKRT. One study showed significantly lower carnitine concentrations at 24 h but not beyond [116], while another study showed that carnitine losses continued during 6 days of follow-up [122].

8. Electrolyte monitoring
  8.1 Routinely monitor serum sodium, chloride, potassium, calcium, phosphorus, magnesium, and bicarbonate (“electrolytes”) throughout the course of AKI. (ungraded)
  8.2 Adjust the frequency of electrolyte monitoring based on laboratory and clinical variables including: trends in electrolyte levels, changes in estimated glomerular filtration rate and urine output, use of or change in KRT prescription or modality, urinary and extrarenal losses of electrolytes and water, adjustments in delivery of electrolytes and fluid to the patient and medications. (ungraded)
9. Electrolyte targets
  9.1 Nutritional electrolyte delivery should be individualized and adjusted in close collaboration with the medical team based on ongoing and anticipated changes in clinical status, medications, and KRT prescription, generally aiming for the normal blood/serum ranges. (ungraded)
  9.2 Adjust nutritional and non-nutritional sodium and water delivery to optimize intravascular volume, with the goal to maintain adequate perfusion and prevent or correct volume overload or depletion. (ungraded)
    9.2.1 Chronically low or high serum sodium values should be corrected gradually to minimize the risk of neurological complications. (ungraded)

*Rationale*: Electrolyte abnormalities are common among patients with sustained AKI [123]. In some cases, KRT is necessary to manage life-threatening electrolyte and acid–base derangements, particularly severe hyperkalemia and acidosis. However, no studies have investigated the optimal frequency at which electrolytes should be monitored in AKI, as it is based on the clinical status of the patient, which can be very labile in children. A patient’s clinical condition and biochemical parameters are usually the primary drivers of fluid and electrolyte management, not nutritional parameters; hence, fluid and electrolyte delivery should be determined in conjunction with the clinical team.

Initial electrolyte monitoring may be required every 8 to 12 h, and occasionally more frequently, depending on the individual patient’s clinical status. Once electrolytes have stabilized, the frequency of laboratory monitoring in the absence of other changes in patient-related variables, including urine output, may be less frequent, though subsequent increases in the frequency of monitoring may be needed due to changes in clinical status, especially initiating or stopping KRT (Supplementary Table 4).

Assessment of serum electrolyte values may be necessary prior to initiating intermittent HD. In patients requiring CKRT, daily electrolytes should be obtained so that the KRT prescription can be adjusted as necessary. Volume status may be a key driver of sodium balance and should be monitored on an ongoing basis. It is also important to consider medication changes when deciding upon appropriate electrolyte monitoring frequency, given that medications may influence electrolytes (Supplementary Table 5).

No studies have specifically investigated the electrolyte needs of children with AKI. It is not possible to provide a general prescription that would be appropriate for every patient given the extreme clinical heterogeneity in this population, including the level of kidney function and whether the patient is receiving KRT. The normal electrolyte requirements for healthy children may be used as an initial framework, but different clinical scenarios (e.g., extrarenal fluid and electrolyte losses), levels of kidney function, urine output, and different dialysis modalities will dictate ongoing adjustments (Table 5).

**Table 5:** Electrolyte requirements in healthy individuals and potential adjustments in patients with AKI

	Healthy children					Patients with AKI*					
	ASPEN		ESPEN			AKI without KRT		iHD	PD	CKRT	Special Considerations
Electrolyte	Infants/children	Adolescents and children > 50 kg	0–18 yr								
Sodium (mEq/kg/d)	2–5	1–2	1–3			↓	↓		↓	↓	Driven mainly by fluid balance
Potassium (mEq/kg/d)	2–4	1–2	1–3			↓	=		=	↑	Can be regulated by dialysis bags
			0–6 months	7–12 months	1–18 yr						
Calcium (mMol/kg/d)°	0.25–2	5–10 mMol/day	0.8–1.5	0.5	0.25–0.4	↑	↑		↑	↑	
Phosphorus (mMol/kg/d)	0.5–2	10–40 mMol/day	0.7–1.3	0.5	0.2–0.7	↓	= ↓		= ↓	↑	Can be regulated by dialysis bags
Magnesium (mMol/kg/d)°	0.15–0.25	5–15 mMol/day	0.1–0.2	0.15	0.1	=	=		=	↑	
Acetate/bicarbonate	As needed to maintain acid–base balance					↑	=		=	↓	

Intended not as a prescription, but rather as a description of what is usually needed in most patients with AKI; for conversion, 1 mMol = 2 mEq; *AKI*, acute kidney injury; *ASPEN*, American Society for Parenteral and Enteral Nutrition; *ESPEN*, European Society for Parenteral and Enteral Nutrition; *KRT*, kidney replacement therapy; *iHD*, intermittent hemodialysis; *PD*, peritoneal dialysis; *CKRT*, continuous kidney replacement therapy

Serum potassium concentrations frequently increase in the setting of AKI due to decreased excretion, and thus intake is typically limited [124]. However, potassium losses in patients with diarrhea can be high, which may require replacement [124]. This may also occur in patients with certain tubular disorders if urine output is still present [125]. Conversely, patients with some disorders (e.g., rhabdomyolysis) or clinical situations (e.g., need for frequent red blood cell transfusions) may develop hyperkalemia and thus require limited intake and/or increased potassium removal via KRT [126]. Potassium is readily removed via KRT and the amount can be adjusted based on the potassium concentration of the dialysis or replacement fluid [127]. Changing the dialysate/replacement potassium concentration, stopping dialysis, and changing dialysis modality may dramatically affect potassium removal. Hence, it is critical that all healthcare providers communicate with each other regarding planned changes in the KRT prescription in order that nutritional potassium intake can be adjusted appropriately.

Most children with AKI develop a metabolic acidosis due to decreased acid excretion, which may be accentuated by certain clinical situations (e.g., diarrhea, lactic acidosis). Children not receiving KRT may require base supplementation [128, 129]. Conversely, gastric losses via emesis or nasogastric losses may cause a metabolic alkalosis. Acid–base balance impacts serum potassium (i.e., decreasing pH tends to increase serum potassium) and ionized calcium (i.e., decreasing pH tends to increase serum ionized calcium). These effects should be considered when correcting acid–base disorders in patients with potassium or calcium disorders (e.g., delay or avoid correcting acidosis with base in a patient with a very low serum potassium or ionized calcium). KRT provides a base to patients which tends to correct metabolic acidosis [130]. The amount of base provided will vary depending on the KRT prescription [131]. Physicians and dietitians should effectively communicate all changes in the KRT prescription that will influence base delivery, including the decision to stop KRT or changes to dialysis modality.

Hyperphosphatemia is common with AKI due to decreased phosphorus excretion, though levels may be low due to decreased intake or intracellular shifts [132, 133]. Decreased phosphorus intake is nevertheless frequently necessary. While KRT removes phosphate, the amount removed is generally limited with intermittent therapies. In contrast, CKRT may remove substantial amounts of phosphate [123, 130, 134, 135]. The amount of phosphate removed can be adjusted in some forms of CKRT [127, 135]. Physicians and dietitians should communicate regarding all changes to the KRT prescription that influence phosphate removal, including the decision to stop KRT or changes to dialysis modality.

In AKI, hypocalcemia may occur through a variety of mechanisms [133]. Hypocalcemia is also common in critically ill patients [132]. Though the amount varies based on dialysis modality and prescription, KRT generally results in a net delivery of calcium to patients. In some forms of CKRT, patients receive a continuous calcium infusion (“drip”) that is adjusted to maintain a normal ionized calcium level. In these situations, the nutritional calcium prescription is not typically adjusted based on the serum calcium level. Hence, it is important that dietitians are aware if a patient is receiving a calcium drip.

Due to decreased excretion, magnesium levels classically increase in AKI, though many critically ill patients develop hypomagnesemia [136–138]. Hence, the nutritional delivery of magnesium is adjusted based on serum levels. KRT results in the net removal of magnesium, with the amount depending on the dialysis modality and prescription [123, 130]. Physicians and dietitians should communicate regarding all changes in the KRT prescription that will influence magnesium removal, including the decision to stop KRT or changes to dialysis modality.

There is no reason to target plasma electrolyte levels differently from the normal range values in healthy children. One exception is the rate at which serum sodium is corrected. Rapid correction of significantly low or high serum sodium values risks neurological complications, so correction should be done gradually [139, 140].

Sodium intake should be adjusted after consideration of both plasma sodium levels and fluid balance. Moreover, water intake has a dramatic effect on plasma sodium values and fluid balance. Hyponatremia in AKI is often due to volume excess, which should be treated by restricting fluid intake rather than by administering additional sodium. Fluid overload is recognized as an independent risk factor for mortality in children with AKI [141]. Sodium restriction is mandatory to mitigate or prevent fluid overload. Thirst is mainly an osmometric process; therefore, a reduction in sodium intake is required when a reduction of water intake is prescribed. Most patients with AKI not treated with KRT will require a restriction of sodium and water intake which, in turn, may limit the provision of optimal nutritional management. The need to restrict fluid and sodium intake may decrease or stop as urine output improves. KRT is very effective at removing sodium and water, often allowing a substantial liberalization of fluid intake. However, hemodynamic considerations may limit fluid removal with intermittent KRT. In contrast, CKRT typically does not require any restrictions of fluid intake since fluid can be removed continuously. However, this may change dramatically when a patient stops CKRT or changes to a different dialysis modality. The physician–dietitian team should communicate regarding the decision to stop KRT or any changes to dialysis modality.

A normal plasma sodium concentration is a reasonable target. However, in case of moderate or profound hyponatremia or hypernatremia, achieving target levels should be approached gradually to avoid serious complications like osmotic demyelinating syndrome (in case of overly rapid correction of hyponatremia) or cerebral edema (when hypernatremia is corrected too fast) [139, 140]. There are rare patients in whom a high-normal or mildly elevated sodium concentration is targeted due to cerebral edema [142].

## Results of the Delphi survey

Thirty responses were received via an electronic Delphi survey, comprising 16 dietitians and 18 pediatric nephrologists across 22 countries. Delphi respondents are listed under Acknowledgements as “Participants in Delphi survey.” The 37 clinical practice recommendation statements received an overall 88% consensus with a “strongly agree” or “agree” response and 11% with a “neutral” response. There were limited numbers of “disagree” with no respondents reporting “strongly disagree” for any statement. All statements met the stipulated 70% or higher level of consensus. Of note, three statements had a high number of “neutral” responses (4.2, 5.5, 7.2) where we suspect this being potentially due to a lack of experience with nutrition support in critically ill children. Taskforce members reviewed comments and agreed that these statements did not require alteration as the GRADE reflected the evidence. Otherwise, minor grammatical adjustments were made to two statements to provide better clarification.

## Summary of recommendations

A summary of recommendations is provided in Table 6.

**Table 6:** Summary of recommendations

Category	Recommendation	Grade
Collaboration	1.1 Ensure close collaboration between the healthcare professionals medical management and those providing the nutritional prescription for the optimal overall care of children with acute kidney injury (AKI).	Level X; Strong recommendation
Nutrition Assessment	2.1 Utilize a validated pediatric nutrition risk screening tool for the assessment of nutritional risk within 48 hours of AKI diagnosis.	Level B; Moderate recommendation
	2.2 Refer any patient found to be at nutritional risk to a dietitian for nutritional assessment.	Level B; Moderate recommendation
	2.3 Repeat nutritional assessments in accordance with the severity of nutritional risk, severity and duration of AKI, and changes in kidney replacement therapy (KRT).	Level D; Weak recommendation
	2.4 Obtain accurate anthropometric measurements as soon as feasible and throughout hospitalization.	Level A, strong recommendation
	2.4.1 Estimate euvolemic weight using accurate trended weight measurements in conjunction with other clinical assessment measures such as fluid balance, blood pressure, physical examination, and available biometric tools (e.g., bioelectrical impedance analysis, mid-upper arm circumference, non-invasive blood volume monitoring).	Level D; weak recommendation
	2.4.2 Measure height or recumbent length for children under 2 years of age. For those unable to stand for accurate height measurement use recumbent length or a surrogate measurement of height.	Level A; strong recommendation
	2.4.3 Measure head circumference for children up to 2 years of age or up to 3 years of age when appropriate centile charts are available.	Level A; strong recommendation
	2.4.4 Assess for muscle wasting by physical assessment and use of biometric tools where available.	Level D; weak recommendation
Oral & Enteral Feeding	3.1 Oral feeding, including breastfeeding, is the preferred method of providing nutrition.	Level X; strong recommendation
	3.2 In critically ill children, consider early initiation (within 48 hours of admission) of supplemental or exclusive enteral tube feeding when oral feeding does not meet nutritional requirements, especially when nutritional intake is likely to remain suboptimal.	Level C; weak recommendation
	3.3 Use whole protein (polymeric) formulas unless otherwise indicated, such as in case of gastrointestinal dysfunction.	Level C; weak recommendation
	3.4 Consider the use of protein and energy dense formulas to achieve nutritional goals within the limits of the fluid allowance and gastrointestinal tolerance; adjust formula density gradually to maximize tolerance.	Level C; moderate recommendation
Parenteral Nutrition	4.1 For children with malnutrition or risk for nutrition deterioration, when oral or enteral nutrition cannot provide all nutritional requirements, initiate supplemental parenteral nutrition (PN).	Level X, moderate recommendation
	4.2 For children without malnutrition or risk for nutrition deterioration, when oral or enteral nutrition cannot provide all nutritional requirements, PN may be withheld for up to 1 week provided micronutrients are delivered.	Level B; moderate recommendation
	4.3 For all children, regardless of nutritional status, receiving KRT that causes significant nutrient losses, initiation of PN before 1 week should be considered when oral or enteral nutrition cannot provide all nutritional requirements.	Level D; weak recommendation
	4.3.1 Pay careful attention when transitioning from PN to enteral feeding in children receiving continuous kidney replacement therapy (CKRT) to ensure provision of optimal nutrition within the fluid allowance.	Level C; moderate recommendation
Energy Requirements	5.1 For non-critically ill children, the initial prescription for energy intake should approximate the suggested dietary intake (SDI) based on euvolemic weight, not measured weight.	Level B; moderate recommendation
	5.2 In the acute phase of critical illness, energy requirements should not exceed the resting energy expenditure.	Level C; weak recommendation
	5.3 In the stable phase and recovery phase of critical illness, the energy prescription must account for energy debt, physical activity, rehabilitation and growth.	Level X; moderate recommendation
	5.4 Modify the energy prescription to account for dialysis-related net gain or loss of energy.	Level C; weak recommendation
	5.5 In critical illness, consider an increased percentage of energy intake from fat to reflect changes in beta-oxidation when parenterally fed.	Level C; weak recommendation
Protein Requirements	6.1 For non-critically ill children, the initial prescription for protein intake should approximate the SDI based on euvolemic weight, not measured weight.	Level B; moderate recommendation
	6.2 For critically ill children, consider the potential need for increased protein intake above the SDI to limit negative protein balance.	Level B; moderate recommendation
	6.3 In children with very elevated blood urea nitrogen levels, especially if progressively worsening, first ensure adequate energy intake; then consider a temporary lowering of protein intake towards the lower end of the SDI.	Level C; moderate recommendation
	6.3.1 Do not persistently compromise protein intake to lower urea nitrogen levels or postpone KRT initiation.	Level X; strong recommendation
	6.4 For all children receiving dialysis, protein prescription needs to be further increased to account for dialysis losses, which are highest in CKRT.	Level C; moderate recommendation
Micronutrient Needs	7.1 In children who are conservatively managed, provide the recommended requirements of vitamins and trace elements for healthy children, with supplementation in case of insufficient intake.	Level D; weak recommendation
	7.2 In children requiring dialysis, consider providing additional supplemental water-soluble vitamins, selenium, copper, zinc and carnitine; either enterally or parenterally.	Level D; weak recommendation
	7.3 Avoid supplemental vitamin A in all children with AKI.	Level B; strong recommendation
	7.4 Evaluate for clinical signs and symptoms of deficiency or excess of vitamins, trace elements and carnitine.	Level C; weak recommendation
	7.5 Do not routinely measure serum concentrations of vitamins, trace elements and carnitine unless there are clinical signs or symptoms of deficiency/toxicity, or when the child is receiving treatment for deficiency or has known toxic concentrations.	Level D; weak recommendation
Electrolyte Monitoring	8.1 Routinely monitor serum sodium, chloride, potassium, calcium, phosphorus, magnesium and bicarbonate (“electrolytes”) throughout the course of AKI.	Ungraded*
	8.2 Adjust the frequency of electrolyte monitoring based on laboratory and clinical variables including: trends in electrolyte levels, changes in estimated glomerular filtration rate and urine output, use of or change in KRT prescription or modality, urinary and extrarenal losses of electrolytes and water, adjustments in delivery of electrolytes and fluid to the patient and medications.	Ungraded*
Electrolyte Targets	9.1 Nutritional electrolyte delivery should be individualized and adjusted in close collaboration with the medical team based on ongoing and anticipated changes in clinical status, medications and KRT prescription, generally aiming for the normal blood/serum ranges.	Ungraded*
	9.2 Adjust nutritional and non-nutritional sodium and water delivery to optimize intravascular volume, with the goal to maintain adequate perfusion and prevent or correct volume overload or depletion.	Ungraded*
	9.2.1 Chronically low or high serum sodium values should be corrected gradually to minimize the risk of neurologic complications.	Ungraded*

^*^A patient’s clinical condition and biochemical parameters are usually the primary drivers of fluid and electrolyte management, not nutritional parameters; hence, fluid and electrolyte delivery should be determined in conjunction with the clinical team

## Research recommendations

We recommend the following areas of study to provide future evidence-based recommendations for the nutritional management of children with AKI.

- Incorporation of dietitians within pediatric AKI-based studies and collaboratives in order to provide optimal nutrition-based metrics that are relevant and meaningful to the growth/development of children and nutrition practice.
- Studies evaluating energy expenditure utilizing indirect calorimetry and assessing the impact of non-nutritive calorie exposure.
- Protein balance studies investigating protein balance in all modalities of acute AKI therapies.
- Studies investigating micronutrient balances in all acute AKI therapies.
- Stu﻿dies ﻿evaluating the effect of nutrition interventions on patient outcomes in all AKI treatment modalities.

### Supplementary Information

Below is the link to the electronic supplementary material.

Supplementary file1 (DOCX 340 KB)

## References

[1] McAlister L, Pugh P, Greenbaum L, Haffner D, Rees L, Anderson C, Desloovere A, Nelms C, Oosterveld M, Paglialonga F, Polderman N, Qizalbash L, Renken-Terhaerdt J, Tuokkola J, Warady B, Walle JV, Shaw V, Shroff R (2020). The dietary management of calcium and phosphate in children with CKD stages 2–5 and on dialysis-clinical practice recommendation from the Pediatric Renal Nutrition Taskforce. Pediatr Nephrol.

[2] Khwaja A (2012). KDIGO clinical practice guidelines for acute kidney injury. Nephron Clin Pract.

[3] Shaw V, Polderman N, Renken-Terhaerdt J, Paglialonga F, Oosterveld M, Tuokkola J, Anderson C, Desloovere A, Greenbaum L, Haffner D, Nelms C, Qizalbash L, Vande Walle J, Warady B, Shroff R, Rees L (2020). Energy and protein requirements for children with CKD stages 2–5 and on dialysis-clinical practice recommendations from the Pediatric Renal Nutrition Taskforce. Pediatr Nephrol.

[4] Burgener AM (2020). Enhancing communication to improve patient safety and to increase patient satisfaction. Health Care Manag (Frederick).

[5] Joosten KF, Hulst JM (2008). Prevalence of malnutrition in pediatric hospital patients. Curr Opin Pediatr.

[6] Castillo A, Santiago MJ, Lopez-Herce J, Montoro S, Lopez J, Bustinza A, Moral R, Bellon JM (2012). Nutritional status and clinical outcome of children on continuous renal replacement therapy: a prospective observational study. BMC Nephrol.

[7] Lion RP, Vega MR, Smith EO, Devaraj S, Braun MC, Bryan NS, Desai MS, Coss-Bu JA, Ikizler TA, Akcan Arikan A (2022). The effect of continuous venovenous hemodiafiltration on amino acid delivery, clearance, and removal in children. Pediatr Nephrol.

[8] Vega MW, Juarez M, Lee JY, Srivaths P, Williams E, Akcan Arikan A (2018). Quality improvement bedside rounding audits enhance protein provision for pediatric patients receiving continuous renal replacement therapy. Pediatr Crit Care Med.

[9] Smith M, Bell C, Vega MW, Tufan Pekkucuksen N, Loftis L, McPherson M, Graf J, Akcan Arikan A (2022). Patient-centered outcomes in pediatric continuous kidney replacement therapy: new morbidity and worsened functional status in survivors. Pediatr Nephrol.

[10] Kyle UG, Akcan-Arikan A, Orellana RA, Coss-Bu JA (2012). Early ICU nutrition support does not meet energy and protein needs in children with acute kidney injury (AKI). Clin Nutr Suppl.

[11] Carvalho-Salemi J, Salemi JL, Wong-Vega MR, Spooner KK, Juarez MD, Beer SS, Canada NL (2018). Malnutrition among hospitalized children in the United States: changing prevalence, clinical correlates, and practice patterns between 2002 and 2011. J Acad Nutr Diet.

[12] Ehwerhemuepha L, Bendig D, Steele C, Rakovski C, Feaster W (2018). The effect of malnutrition on the risk of unplanned 7-day readmission in pediatrics. Hosp Pediatr.

[13] Lezo A, Povero M, Pradelli L, Nigro E, Plazzotta C, Lagazio C (2021). Assessing the effect of nutrition therapy on rehospitalization rate in malnourished pediatric patients with chronic diseases. J Parenter Enteral Nutr.

[14] Secker DJ, Jeejeebhoy KN (2007). Subjective Global Nutritional Assessment for children. Am J Clin Nutr.

[15] Li C, Xu L, Guan C, Zhao L, Luo C, Zhou B, Zhang X, Wang J, Zhao J, Huang J, Li D, Luan H, Man X, Che L, Wang Y, Zhang H, Xu Y (2020). Malnutrition screening and acute kidney injury in hospitalised patients: a retrospective study over a 5-year period from China. Br J Nutr.

[16] Klanjsek P, Pajnkihar M, Marcun Varda N, Povalej Brzan P (2019) Screening and assessment tools for early detection of malnutrition in hospitalised children: a systematic review of validation studies. BMJ Open 9:e025444. 10.1136/bmjopen-2018-025444 10.1136/bmjopen-2018-025444 PMC6549612 31138579

[17] Joosten KF, Hulst JM (2014). Nutritional screening tools for hospitalized children: methodological considerations. Clin Nutr.

[18] Becker PJ, Gunnell Bellini S, Wong Vega M, Corkins MR, Spear BA, Spoede E, Hoy MK, Piemonte TA, Rozga M (2020). Validity and reliability of pediatric nutrition screening tools for hospital, outpatient, and community settings: a 2018 evidence analysis center systematic review. J Acad Nutr Diet.

[19] Zamberlan P, Feferbaum RAPOP, Doria Filho U, Brunow de Carvalho W, Figueiredo Delgado A (2019). Bioelectrical impedance phase angle and morbidity and mortality in critically ill children. Nutr Clin Pract.

[20] Bloomfield R, Steel E, MacLennan G, Noble DW (2006). Accuracy of weight and height estimation in an intensive care unit: implications for clinical practice and research. Crit Care Med.

[21] Pace A, Zobel A, Gearman L, Seitzer D, Larson-Nath C, Somani A (2021). Improving the rate of anthropometric measurements in the pediatric intensive care unit. Nutr Clin Pract.

[22] Nelms CL, Shaw V, Greenbaum LA, Anderson C, Desloovere A, Haffner D, Oosterveld MJS, Paglialonga F, Polderman N, Qizalbash L, Rees L, Renken-Terhaerdt J, Tuokkola J, Vande Walle J, Shroff R, Warady BA (2021). Assessment of nutritional status in children with kidney diseases-clinical practice recommendations from the Pediatric Renal Nutrition Taskforce. Pediatr Nephrol.

[23] Prado CM, Heymsfield SB (2014). Lean tissue imaging: a new era for nutritional assessment and intervention. J Parenter Enteral Nutr.

[24] Kyle UG, Earthman CP, Pichard C, Coss-Bu JA (2015). Body composition during growth in children: limitations and perspectives of bioelectrical impedance analysis. Eur J Clin Nutr.

[25] Dasgupta I, Keane D, Lindley E, Shaheen I, Tyerman K, Schaefer F, Wühl E, Müller MJ, Bosy-Westphal A, Fors H, Dahlgren J, Chamney P, Wabel P, Moissl U (2018). Validating the use of bioimpedance spectroscopy for assessment of fluid status in children. Pediatr Nephrol.

[26] Hise A, Gonzalez MC (2018). Assessment of hydration status using bioelectrical impedance vector analysis in critical patients with acute kidney injury. Clin Nutr.

[27] Simoni P, Guglielmi R, Aparisi Gomez MP (2020) Imaging of body composition in children. Quant Imaging Med Surg 10:1661–1671. 10.21037/qims.2020.04.06 10.21037/qims.2020.04.06 PMC7378095 32742959

[28] de Figueiredo RS, Nogueira RJN, Springer AMM, Melro EC, Campos NB, Batalha RE, Brandão MB, de Souza TH (2021). Sarcopenia in critically ill children: a bedside assessment using point-of-care ultrasound and anthropometry. Clin Nutr.

[29] Ong C, Lee JH, Leow MKS, Puthucheary ZA (2017). Skeletal muscle ultrasonography in nutrition and functional outcome assessment of critically ill children: experience and insights from pediatric disease and adult critical care studies. J Parenter Enteral Nutr.

[30] Wang F, Wang C, Shi J, Shan Y, Miao H, Sun T, Zhou Y, Zhang Y (2021). Lung ultrasound score assessing the pulmonary edema in pediatric acute respiratory distress syndrome received continuous hemofiltration therapy: a prospective observational study. BMC Pulm Med.

[31] Torterüe X, Dehoux L, Macher MA, Niel O, Kwon T, Deschênes G, Hogan J (2017). Fluid status evaluation by inferior vena cava diameter and bioimpedance spectroscopy in pediatric chronic hemodialysis. BMC Nephrol.

[32] Stephens K, Escobar A, Jennison EN, Vaughn L, Sullivan R, Abdel-Rahman S (2018). Evaluating mid-upper arm circumference Z-score as a determinant of nutrition status. Nutr Clin Pract.

[33] Addo OY, Himes JH, Zemel BS (2017). Reference ranges for midupper arm circumference, upper arm muscle area, and upper arm fat area in US children and adolescents aged 1–20 y. Am J Clin Nutr.

[34] de Abreu AM, Wilvert LC, Wazlawik E (2020). Comparison of body mass index, skinfold thickness, and bioelectrical impedance analysis with dual-energy X-ray absorptiometry in hemodialysis patients. Nutr Clin Pract.

[35] Vermilyea S, Slicker J, El-Chammas K, Sultan M, Dasgupta M, Hoffmann RG, Wakeham M, Goday PS (2013). Subjective global nutritional assessment in critically ill children. J Parenter Enteral Nutr.

[36] Baker JP, Detsky AS, Wesson DE, Wolman SL, Stewart S, Whitewell J, Langer B, Jeejeebhoy KN (1982). Nutritional assessment: a comparison of clinical judgement and objective measurements. N Engl J Med.

[37] Selewski DT, Cornell TT, Lombel RM, Blatt NB, Han YY, Mottes T, Kommareddi M, Kershaw DB, Shanley TP, Heung M (2011). Weight-based determination of fluid overload status and mortality in pediatric intensive care unit patients requiring continuous renal replacement therapy. Intensive Care Med.

[38] Jain SR, Smith L, Brewer ED, Goldstein SL (2001). Non-invasive intravascular monitoring in the pediatric hemodialysis population. Pediatr Nephrol.

[39] Wong Vega M, Beer S, Juarez M, Srivaths PR (2019). Malnutrition risk in hospitalized children: a descriptive study of malnutrition-related characteristics and development of a pilot pediatric risk-assessment tool. Nutr Clin Pract.

[40] Zamberlan P, Delgado AF, Leone C, Feferbaum R, Okay TS (2011). Nutrition therapy in a pediatric intensive care unit: indications, monitoring, and complications. J Parenter Enteral Nutr.

[41] Apostolou A, Printza N, Karagiozoglou-Lampoudi T, Dotis J, Papachristou F (2014). Nutrition assessment of children with advanced stages of chronic kidney disease-a single center study. Hippokratia.

[42] Irving SY, Seiple S, Nagle M, Falk S, Mascarenhas M, Srinivasan V (2015). Perceived barriers to anthropometric measurements in critically ill children. Am J Crit Care.

[43] Ford N, Hargreaves S, Shanks L (2012). Mortality after fluid bolus in children with shock due to sepsis or severe infection: a systematic review and meta-analysis. PLoS One.

[44] Dale NM, Myatt M, Prudhon C, Briend A (2013). Using mid-upper arm circumference to end treatment of severe acute malnutrition leads to higher weight gains in the most malnourished children. PLoS One.

[45] Park KH, Shin JH, Hwang JH, Kim SH (2017). Utility of volume assessment using bioelectrical impedance analysis in critically ill patients receiving continuous renal replacement therapy: a prospective observational study. Korean J Crit Care Med.

[46] Schapka E, Gee J, Cyrus JW, Goldstein G, Greenfield K, Marinello M, Karam O (2021) Lung ultrasound versus chest X-ray for the detection of fluid overload in critically ill children: a systematic review. J Pediatr Intensive Care 11:177–182. 10.1055/s-0041-1725123 10.1055/s-0041-1725123 PMC9345674 35928036

[47] Johnson RW, Ng KWP, Dietz AR, Hartman ME, Baty JD, Hasan N, Zaidman CM, Shoykhet M (2018). Muscle atrophy in mechanically-ventilated critically ill children. PLoS One.

[48] Hoffmann RM, Ariagno KA, Pham IV, Barnewolt CE, Jarrett DY, Mehta NM, Kantor DB (2021). Ultrasound assessment of quadriceps femoris muscle thickness in critically ill children. Pediatr Crit Care Med.

[49] Tume LN, Valla FV, Joosten K, Jotterand Chaparro C, Latten L, Marino LV, Macleod I, Moullet C, Pathan N, Rooze S, van Rosmalen J, Verbruggen S (2020). Nutritional support for children during critical illness: European Society of Pediatric and Neonatal Intensive Care (ESPNIC) metabolism, endocrine and nutrition section position statement and clinical recommendations. Intensive Care Med.

[50] Mehta NM, Bechard LJ, Zurakowski D, Duggan CP, Heyland DK (2015). Adequate enteral protein intake is inversely associated with 60-d mortality in critically ill children: a multicenter, prospective, cohort study. Am J Clin Nutr.

[51] Andreas NJ, Kampmann B, Mehring Le-Doare K (2015). Human breast milk: a review on its composition and bioactivity. Early Hum Dev.

[52] Horta BL, Victoria CG (2013) Long-term effects of breastfeeding: a systematic review. World Health Organization, Geneva. https://apps.who.int/iris/bitstream/handle/10665/79198/97892?sequence=1

[53] Hubbard GP, Fry C, Sorensen K, Casewell C, Collins L, Cunjamalay A, Simpson M, Wall A, Van Wyk E, Ward M, Hallowes S, Duggan H, Robison J, Gane H, Pope L, Clark J, Stratton RJ (2020). Energy-dense, low-volume paediatric oral nutritional supplements improve total nutrient intake and increase growth in paediatric patients requiring nutritional support: results of a randomised controlled pilot trial. Eur J Pediatr.

[54] Brierley-Hobson S, Clarke G, O'Keeffe V (2019). Safety and efficacy of volume-based feeding in critically ill, mechanically ventilated adults using the 'protein & energy requirements fed for every critically ill patient every time' (PERFECT) protocol: a before-and-after study. Crit Care.

[55] Mehta NM, Skillman HE, Irving SY, Coss-Bu JA, Vermilyea S, Farrington EA, McKeever L, Hall AM, Goday PS, Braunschweig C (2017). Guidelines for the provision and assessment of nutrition support therapy in the pediatric critically ill patient: society of critical care medicine and American society for parenteral and enteral nutrition. J Parenter Enteral Nutr.

[56] López-Herce J, Santiago MJ, Sánchez C, Mencía S, Carrillo A, Vigil D (2008). Risk factors for gastrointestinal complications in critically ill children with transpyloric enteral nutrition. Eur J Clin Nutr.

[57] Zhang J, Ankawi G, Sun J, Digvijay K, Yin Y, Rosner MH, Ronco C (2018). Gut-kidney crosstalk in septic acute kidney injury. Crit Care.

[58] Wong Vega M, Juarez Calderon M, Tufan Pekkucuksen N, Srivaths P, Akcan Arikan A (2019). Feeding modality is a barrier to adequate protein provision in children receiving continuous renal replacement therapy (CRRT). Pediatr Nephrol.

[59] Fivez T, Kerklaan D, Mesotten D, Verbruggen S, Wouters PJ, Vanhorebeek I, Debaveye Y, Vlasselaers D, Desmet L, Casaer MP, Garcia Guerra G, Hanot J, Joffe A, Tibboel D, Joosten K, Van den Berghe G (2016). Early versus late parenteral nutrition in critically ill children. N Engl J Med.

[60] Vanhorebeek I, Verbruggen S, Casaer MP, Gunst J, Wouters PJ, Hanot J, Guerra GG, Vlasselaers D, Joosten K, Van den Berghe G (2017). Effect of early supplemental parenteral nutrition in the paediatric ICU: a preplanned observational study of post-randomisation treatments in the PEPaNIC trial. Lancet Respir Med.

[61] Greathouse KC, Sakellaris KT, Tumin D, Katsnelson J, Tobias JD, Hayes D, Yates AR (2018). Impact of early initiation of enteral nutrition on survival during pediatric extracorporeal membrane oxygenation. J Parenter Enteral Nutr.

[62] Bechard LJ, Staffa SJ, Zurakowski D, Mehta NM (2021). Time to achieve delivery of nutrition targets is associated with clinical outcomes in critically ill children. Am J Clin Nutr.

[63] Levine B, Mizushima N, Virgin HW (2011). Autophagy in immunity and inflammation. Nature.

[64] Joosten KF, Kerklaan D, Verbruggen SC (2016). Nutritional support and the role of the stress response in critically ill children. Curr Opin Clin Nutr Metab Care.

[65] Preiser JC, Ichai C, Orban JC, Groeneveld AB (2014). Metabolic response to the stress of critical illness. Br J Anaesth.

[66] McCowen KC, Malhotra A, Bistrian BR (2001). Stress-induced hyperglycemia. Crit Care Clin.

[67] Puthucheary ZA, Rawal J, McPhail M, Connolly B, Ratnayake G, Chan P, Hopkinson NS, Phadke R, Dew T, Sidhu PS, Velloso C, Seymour J, Agley CC, Selby A, Limb M, Edwards LM, Smith K, Rowlerson A, Rennie MJ, Moxham J, Harridge SD, Hart N, Montgomery HE (2013). Acute skeletal muscle wasting in critical illness. JAMA.

[68] Parry SM, Puthucheary ZA (2015). The impact of extended bed rest on the musculoskeletal system in the critical care environment. Extrem Physiol Med.

[69] Joosten KFM, Eveleens RD, Verbruggen S (2019). Nutritional support in the recovery phase of critically ill children. Curr Opin Clin Nutr Metab Care.

[70] Schofield WN (1985). Predicting basal metabolic rate, new standards and review of previous work. Hum Nutr Clin Nutr.

[71] (1985) Energy and protein requirements. Report of a joint FAO/WHO/UNU Expert Consultation. World Health Organ Tech Rep Ser 724:1–206. https://www.ncbi.nlm.nih.gov/pubmed/3937340 3937340

[72] Havalad S, Quaid MA, Sapiega V (2006). Energy expenditure in children with severe head injury: lack of agreement between measured and estimated energy expenditure. Nutr Clin Pract.

[73] Meyer R, Kulinskaya E, Briassoulis G, Taylor RM, Cooper M, Pathan N, Habibi P (2012). The challenge of developing a new predictive formula to estimate energy requirements in ventilated critically ill children. Nutr Clin Pract.

[74] White MS, Shepherd RW, McEniery JA (2000). Energy expenditure in 100 ventilated, critically ill children: improving the accuracy of predictive equations. Crit Care Med.

[75] van der Kuip M, de Meer K, Westerterp KR, Gemke RJ (2007). Physical activity as a determinant of total energy expenditure in critically ill children. Clin Nutr.

[76] Fiaccadori E, Sabatino A, Barazzoni R, Carrero JJ, Cupisti A, De Waele E, Jonckheer J, Singer P, Cuerda C (2021). ESPEN guideline on clinical nutrition in hospitalized patients with acute or chronic kidney disease. Clin Nutr.

[77] Jonckheer J, Van Hoorn A, Oshima T, De Waele E (2022). Bioenergetic balance of continuous venovenous hemofiltration, a retrospective analysis. Nutrients.

[78] Egi M, Morimatsu H, Toda Y, Matsusaki T, Suzuki S, Shimizu K, Iwasaki T, Takeuchi M, Bellomo R, Morita K (2008). Hyperglycemia and the outcome of pediatric cardiac surgery patients requiring peritoneal dialysis. Int J Artif Organs.

[79] Vande Walle J, Raes A, Dehoorne J, Mauel R, Dejaeghere A, Matthys D (2004) Combined amino-acid and glucose peritoneal dialysis solution for children with acute renal failure. Adv Perit Dial 20:226–230. https://www.ncbi.nlm.nih.gov/pubmed/15384832 15384832

[80] Coss-Bu JA, Klish WJ, Walding D, Stein F, Smith EO, Jefferson LS (2001). Energy metabolism, nitrogen balance, and substrate utilization in critically ill children. Am J Clin Nutr.

[81] Hellerman M, Sabatino A, Theilla M, Kagan I, Fiaccadori E, Singer P (2019). Carbohydrate and lipid prescription, administration, and oxidation in critically ill patients with acute kidney injury: a post hoc analysis. J Ren Nutr.

[82] Maxvold NJ, Smoyer WE, Custer JR, Bunchman TE (2000). Amino acid loss and nitrogen balance in critically ill children with acute renal failure: a prospective comparison between classic hemofiltration and hemofiltration with dialysis. Crit Care Med.

[83] Zappitelli M, Goldstein SL, Symons JM, Somers MJG, Baum MA, Brophy PD, Blowey D, Fortenberry JD, Chua AN, Flores FX, Benfield MR, Alexander SR, Askenazi D, Hackbarth R, Bunchman TE (2008). Protein and calorie prescription for children and young adults receiving continuous renal replacement therapy: a report from the prospective pediatric continuous renal replacement therapy registry group. Crit Care Med.

[84] Sabatino A, Regolisti G, Maggiore U, Fiaccadori E (2014). Protein/energy debt in critically ill children in the pediatric intensive care unit: acute kidney injury as a major risk factor. J Ren Nutr.

[85] Miller RL, Taylor WR, Gentry W, Day AT, Gazzaniga AB (1983) Indirect calorimetry in postoperative patients with acute renal failure. Am Surg 49:494–499. https://www.ncbi.nlm.nih.gov/pubmed/6625361 6625361

[86] Kritmetapak K, Peerapornratana S, Srisawat N, Somlaw N, Lakananurak N, Dissayabutra T, Phonork C, Leelahavanichkul A, Tiranathanagul K, Susantithapong P, Loaveeravat P, Suwachittanont N, Wirotwan TO, Praditpornsilpa K, Tungsanga K, Eiam-Ong S, Kittiskulnam P (2016). The impact of macro-and micronutrients on predicting outcomes of critically ill patients requiring continuous renal replacement therapy. PLoS One.

[87] Ganesan MV, Annigeri RA, Shankar B, Rao BS, Prakash KC, Seshadri R, Mani MK (2009). The protein equivalent of nitrogen appearance in critically ill acute renal failure patients undergoing continuous renal replacement therapy. J Ren Nutr.

[88] Wang XH, Mitch WE (2014). Mechanisms of muscle wasting in chronic kidney disease. Nat Rev Nephrol.

[89] Uauy RD, Hogg RJ, Brewer ED, Reisch JS, Cunningham C, Holliday MA (1994). Dietary protein and growth in infants with chronic renal insufficiency: a report from the Southwest Pediatric Nephrology Study Group and the University of California, San Francisco. Pediatr Nephrol.

[90] Wong CS, Gipson DS, Gillen DL, Emerson S, Koepsell T, Sherrard DJ, Watkins SL, Stehman-Breen C (2000). Anthropometric measures and risk of death in children with end-stage renal disease. Am J Kidney Dis.

[91] Quan A, Baum M (1996). Protein losses in children on continuous cycler peritoneal dialysis. Pediatr Nephrol.

[92] Edefonti A, Picca M, Damiani B, Loi S, Ghio L, Giani M, Consalvo G, Grassi MR (1999). Dietary prescription based on estimated nitrogen balance during peritoneal dialysis. Pediatr Nephrol.

[93] National Kidney Foundation (2009). KDOQI clinical practice guideline for nutrition in children with CKD: 2008 update. Am J Kidney Dis.

[94] Zappitelli M, Juarez M, Castillo L, Coss-Bu J, Goldstein SL (2009). Continuous renal replacement therapy amino acid, trace metal and folate clearance in critically ill children. Intensive Care Med.

[95] Ni C, Cao J, Li D, Wu W, Cao L, Zhu C (2020) Parenteral nutrition effects of omega-3 fatty acids on C-reactive protein, high-density lipoprotein, lymphocyte characteristics and the treatment of critically ill patients. Cell Mol Biol (Noisy-le-grand) 66:52–56. https://www.ncbi.nlm.nih.gov/pubmed/32538747 32538747

[96] Harshman LA, Lee-Son K, Jetton JG (2018). Vitamin and trace element deficiencies in the pediatric dialysis patient. Pediatr Nephrol.

[97] Dao DT, Anez-Bustillos L, Cho BS, Li Z, Puder M, Gura KM (2017). Assessment of micronutrient status in critically ill children: challenges and opportunities. Nutrients.

[98] Marino LV, Valla FV, Beattie RM, Verbruggen S (2020). Micronutrient status during paediatric critical illness: a scoping review. Clin Nutr.

[99] Joyce T, Court Brown F, Wallace D, Reid CJD, Sinha MD (2018). Trace element and vitamin concentrations in paediatric dialysis patients. Pediatr Nephrol.

[100] Blomhoff R (1994) Overview of vitamin A metabolism and function in vitamin A in health and disease. eBook, 1st edn. Marcel Dekker, New York, p 1–35. 10.1201/9781482277562

[101] Manickavasagar B, McArdle AJ, Yadav P, Shaw V, Dixon M, Blomhoff R, Connor GO, Rees L, Ledermann S, Van't Hoff W, Shroff R (2015). Hypervitaminosis A is prevalent in children with CKD and contributes to hypercalcemia. Pediatr Nephrol.

[102] EFSA Nda Panel (EFSA Panel on Dietetic Products Nutrition and Allergies) (2014). Scientific opinion on dietary reference values for chromium. EFSA J.

[103] EFSA Nda Panel (EFSA Panel on Dietetic Products Nutrition and Allergies) (2013). Scientific opinion on dietary reference values for manganese. EFSA J.

[104] (2009) KDOQI clinical practice guideline for nutrition in children with CKD: 2008 update. Executive summary. Am J Kidney Dis 53:S11-104. 10.1053/j.ajkd.2008.11.017 10.1053/j.ajkd.2008.11.017 19231749

[105] Ikizler TA, Burrowes JD, Byham-Gray LD, Campbell KL, Carrero JJ, Chan W, Fouque D, Friedman AN, Ghaddar S, Goldstein-Fuchs DJ, Kaysen GA, Kopple JD, Teta D, Yee-Moon Wang A, Cuppari L (2020). KDOQI clinical practice guideline for nutrition in CKD: 2020 update. Am J Kidney Dis.

[106] Sgambat K, Moudgil A (2016). Carnitine deficiency in children receiving continuous renal replacement therapy. Hemodial Int.

[107] Honore PM, Spapen HD, Marik P, Boer W, Oudemans-van Straaten H (2020). Dosing vitamin C in critically ill patients with special attention to renal replacement therapy: a narrative review. Ann Intensive Care.

[108] Warady BA, Kriley M, Alon U, Hellerstein S (1994). Vitamin status of infants receiving long-term peritoneal dialysis. Pediatr Nephrol.

[109] Wei Q, Dong Z (2020). The yin and yang of retinoic acid signaling in kidney diseases. J Clin Invest.

[110] Oh WC, Mafrici B, Rigby M, Harvey D, Sharman A, Allen JC, Mahajan R, Gardner DS, Devonald MAJ (2019). Micronutrient and amino acid losses during renal replacement therapy for acute kidney injury. Kidney Int Rep.

[111] Jonckheer J, Vergaelen K, Spapen H, Malbrain M, De Waele E (2019). Modification of nutrition therapy during continuous renal replacement therapy in critically ill pediatric patients: a narrative review and recommendations. Nutr Clin Pract.

[112] Maynar Moliner J, Honore PM, Sanchez-Izquierdo Riera JA, Herrera Gutierrez M, Spapen HD (2012). Handling continuous renal replacement therapy-related adverse effects in intensive care unit patients: the dialytrauma concept. Blood Purif.

[113] Pasko DA, Churchwell MD, Btaiche IF, Jain JC, Mueller BA (2009). Continuous venovenous hemodiafiltration trace element clearance in pediatric patients: a case series. Pediatr Nephrol.

[114] Mishra OP, Pooniya V, Ali Z, Upadhyay RS, Prasad R (2008). Antioxidant status of children with acute renal failure. Pediatr Nephrol.

[115] Tonelli M, Wiebe N, Bello A, Field CJ, Gill JS, Hemmelgarn BR, Holmes DT, Jindal K, Klarenbach SW, Manns BJ, Thadhani R, Kinniburgh D (2018). Concentrations of trace elements and clinical outcomes in hemodialysis patients: a prospective cohort study. Clin J Am Soc Nephrol.

[116] Ostermann M, Summers J, Lei K, Card D, Harrington DJ, Sherwood R, Turner C, Dalton N, Peacock J, Bear DE (2020). Micronutrients in critically ill patients with severe acute kidney injury - a prospective study. Sci Rep.

[117] Berger MM, Soguel L, Shenkin A, Revelly JP, Pinget C, Baines M, Chiolero RL (2008). Influence of early antioxidant supplements on clinical evolution and organ function in critically ill cardiac surgery, major trauma, and subarachnoid hemorrhage patients. Crit Care.

[118] Evans AM, Faull RJ, Nation RL, Prasad S, Elias T, Reuter SE, Fornasini G (2004). Impact of hemodialysis on endogenous plasma and muscle carnitine levels in patients with end-stage renal disease. Kidney Int.

[119] Berger MM, Broman M, Forni L, Ostermann M, De Waele E, Wischmeyer PE (2021). Nutrients and micronutrients at risk during renal replacement therapy: a scoping review. Curr Opin Crit Care.

[120] Evans A (2003). Dialysis-related carnitine disorder and levocarnitine pharmacology. Am J Kidney Dis.

[121] Sgambat K, Clauss S, Moudgil A (2021). Effect of levocarnitine supplementation on myocardial strain in children with acute kidney injury receiving continuous kidney replacement therapy: a pilot study. Pediatr Nephrol.

[122] Lumlertgul N, Bear DE, Ostermann M (2020). Clearance of micronutrients during continuous renal replacement therapy. Crit Care.

[123] Finkel KW, Podoll AS (2009). Complications of continuous renal replacement therapy. Semin Dial.

[124] Eliacik E, Yildirim T, Sahin U, Kizilarslanoglu C, Tapan U, Aybal-Kutlugun A, Hascelik G, Arici M (2015). Potassium abnormalities in current clinical practice: frequency, causes, severity and management. Med Princ Pract.

[125] Zieg J, Gonsorcikova L, Landau D (2016). Current views on the diagnosis and management of hypokalaemia in children. Acta Paediatr.

[126] Młynarska E, Krzemińska J, Wronka M, Franczyk B, Rysz J (2022). Rhabdomyolysis-induced AKI (RIAKI) including the role of COVID-19. Int J Mol Sci.

[127] Cho A, Lee YK, Park HC (2020). Impact of electrolyte-rich dialysate during continuous renal replacement therapy on serum phosphate and potassium in ICU patients. PLoS One.

[128] Evans L, Rhodes A, Alhazzani W, Antonelli M, Coopersmith CM, French C, Machado FR, McIntyre L, Ostermann M, Prescott HC, Schorr C, Simpson S, Wiersinga WJ, Alshamsi F, Angus DC, Arabi Y, Azevedo L, Beale R, Beilman G, Belley-Cote E, Burry L, Cecconi M, Centofanti J, Coz Yataco A, De Waele J, Dellinger RP, Doi K, Du B, Estenssoro E, Ferrer R, Gomersall C, Hodgson C, Hylander Møller M, Iwashyna T, Jacob S, Kleinpell R, Klompas M, Koh Y, Kumar A, Kwizera A, Lobo S, Masur H, McGloughlin S, Mehta S, Mehta Y, Mer M, Nunnally M, Oczkowski S, Osborn T, Papathanassoglou E, Perner A, Puskarich M, Roberts J, Schweickert W, Seckel M, Sevransky J, Sprung CL, Welte T, Zimmerman J, Levy M (2021) Surviving sepsis campaign: international guidelines for management of sepsis and septic shock 2021. Crit Care Med 49:e1063-e1143. 10.1097/ccm.0000000000005337

[129] Jaber S, Paugam C, Futier E, Lefrant JY, Lasocki S, Lescot T, Pottecher J, Demoule A, Ferrandière M, Asehnoune K, Dellamonica J, Velly L, Abback PS, de Jong A, Brunot V, Belafia F, Roquilly A, Chanques G, Muller L, Constantin JM, Bertet H, Klouche K, Molinari N, Jung B (2018). Sodium bicarbonate therapy for patients with severe metabolic acidaemia in the intensive care unit (BICAR-ICU): a multicentre, open-label, randomised controlled, phase 3 trial. Lancet.

[130] Macedo E, Mehta RL (2016). Continuous dialysis therapies: core curriculum 2016. Am J Kidney Dis.

[131] Baldwin I, Naka T, Koch B, Fealy N, Bellomo R (2007). A pilot randomised controlled comparison of continuous veno-venous haemofiltration and extended daily dialysis with filtration: effect on small solutes and acid-base balance. Intensive Care Med.

[132] Jung SY, Kim H, Park S, Jhee JH, Yun HR, Kim H, Kee YK, Yoon CY, Oh HJ, Chang TI, Park JT, Yoo TH, Kang SW, Lee H, Kim DK, Han SH (2016). Electrolyte and mineral disturbances in septic acute kidney injury patients undergoing continuous renal replacement therapy. Medicine (Baltimore).

[133] Leaf DE, Christov M (2019). Dysregulated mineral metabolism in AKI. Semin Nephrol.

[134] Busch RA, Curtis CS, Kight CE, Leverson GE, Ma Y, Maursetter L, Kudsk KA (2017). An institutional change in continuous renal replacement therapy: nutrition support team resolves resultant severe hypophosphatemia. Nutr Clin Pract.

[135] Heung M, Mueller BA (2018). Prevention of hypophosphatemia during continuous renal replacement therapy-an overlooked problem. Semin Dial.

[136] Morooka H, Tanaka A, Kasugai D, Ozaki M, Numaguchi A, Maruyama S (2022). Abnormal magnesium levels and their impact on death and acute kidney injury in critically ill children. Pediatr Nephrol.

[137] Martin KJ, González EA, Slatopolsky E (2009). Clinical consequences and management of hypomagnesemia. J Am Soc Nephrol.

[138] Di Mario F, Regolisti G, Greco P, Maccari C, Superchi E, Morabito S, Pistolesi V, Fiaccadori E (2021). Prevention of hypomagnesemia in critically ill patients with acute kidney injury on continuous kidney replacement therapy: the role of early supplementation and close monitoring. J Nephrol.

[139] Adrogué HJ, Tucker BM, Madias NE (2022). Diagnosis and management of hyponatremia: a review. JAMA.

[140] Sterns RH (2015). Disorders of plasma sodium–causes, consequences, and correction. N Engl J Med.

[141] Bhaskar P, Dhar AV, Thompson M, Quigley R, Modem V (2015). Early fluid accumulation in children with shock and ICU mortality: a matched case-control study. Intensive Care Med.

[142] Fülöp T, Zsom L, Rodríguez RD, Chabrier-Rosello JO, Hamrahian M, Koch CA (2019). Therapeutic hypernatremia management during continuous renal replacement therapy with elevated intracranial pressures and respiratory failure. Rev Endocr Metab Disord.

