# Supplementary material for: Nutritional management of children with acute kidney injury—clinical practice recommendations from the Pediatric Renal Nutrition Taskforce
Source: Pediatr Nephrol. 2023 Mar 20;38(11):3559–80. doi: 10.1007/s00467-023-05884-3 (PMC10514117; doi:10.1007/s00467-023-05884-3)
Supplement: Supplementary file 1 — Supplementary file1 (DOCX 340 KB) [file 467_2023_5884_MOESM1_ESM.docx]

SUPPLEMENTARY MATERIAL

**Supplementary Table 1:** Search terms strategy used in the literature review for MEDLINE, EMBASE, Cochrane and CINAHL for Nutritional management of children with AKI

| 1 | **Acute kidney injury** | **Acute renal failure** | **Renal replacement therapy** | **CRRT** | **CKRT** |  |
| --- | --- | --- | --- | --- | --- | --- |
| **2** | **Calorie** | **Protein** | **Energy** | **Nutrient** | **Fat** | **Minerals** |
|  | **Electrolyte** | **Trace elements** | **Amino acid** | **Carbohydrate** | **Vitamins** | **Nutrition support** |
|  | **Feeding** | **Malnutrition** | **Total parenteral nutrition** | **Nutrition assessment** | **Nutrition therapy** | **Enteral nutrition** |

**Search methods**:

- Electronic search using: Medline, PubMed, Embase, Cochrane library, Cinahl, manual searching (via OVID online database)
- 1980 – 2021, English language
- Reference list from review articles and clinical practice guidelines (including KDOQI, KDIGO, ASPEN, ESPEN, ESPNIC)

**Supplementary Table 2:** American Academy of Pediatrics grading


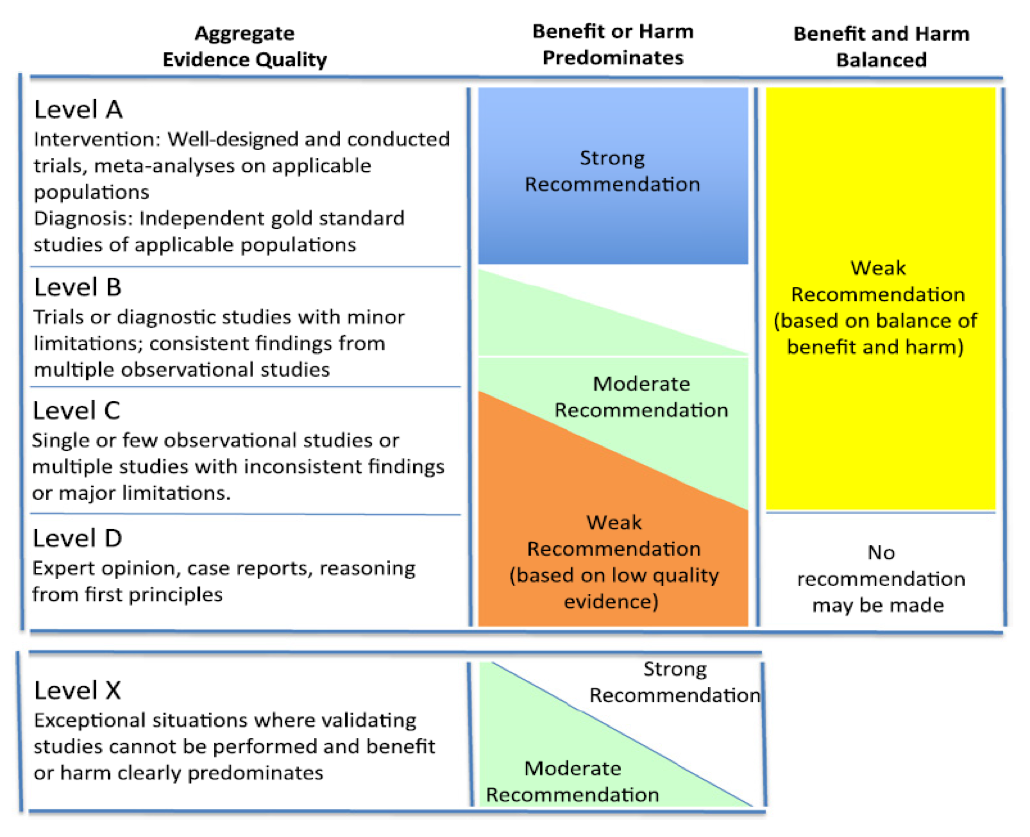


**Supplementary Table 3. Repletion dosing for vitamin and trace mineral deficiencies in the general pediatric population**

| **Micronutrient** | **Repletion Dosing Recommendation** |
| --- | --- |
| Thiamine (B1) | Children: 10-25 mg/day parenterally x 1-2 week, followed by 5 mg PO for 6 weeks[143] |
| Pyridoxine (B6) | 5-25 mg/day of oral pyridoxine x 3 weeks, followed by 2.5 – 5 mg/day orally via multivitamin |
| Folate (B9) | Deficiency in infants: 0.1 mg/kg/day oral administration daily  Deficiency in children: 1.0 mg oral administration daily, followed by 0.1 to 0.5 mg daily maintenance dosing. |
| Vitamin C | 100 mg orally, intramuscularly, or intravenously 3x per day x 1 week, followed by 100 mg daily[144] |
| Zinc | Supplementation trail with 1 mg/kg; can increase to 2 mg/kg two - three times a day[145] |
| Selenium | 2 mcg/kg/day IV selenium for repletion followed by 1 mcg/kg/day as maintenance therapy for goal of serum level of 50 – 150 mcg/L[146] |
| Table adapted from Harshman *et al*[80] Dosing not otherwise established in pediatric CKD/ESKD  PO, per os; CKD, chronic kidney disease; ESKD, end stage kidney disease | |

**Additional References Pertaining to Supplementary Table 3**

143. Sachdev HPS, Shah D (2020) Vitamin B Complex Deficiencies and Excess. In: Kliegman R (ed) Textbook of pediatrics. Elsevier, Philadelphia, PA, pp 365-373.e361.

144. Weinstein M, Babyn P, Zlotkin S (2001) An orange a day keeps the doctor away: scurvy in the year 2000. Pediatrics 108:E55. <https://doi.org/10.1542/peds.108.3.e55>.

145. Hotz C, Brown K (2001) Identifying populations at risk of zinc deficiency: the use of supplementation trials. Nutr Rev 59:80-84.

146. Vinton NE, Dahlstrom KA, Strobel CT, Ament ME (1987) Macrocytosis and pseudoalbinism: manifestations of selenium deficiency. J Pediatr 111:711-717. https://doi.org/10.1016/s0022-3476(87)80247-0.

**Supplementary Table 4: Indications for increased monitoring of electrolytes**

| **Indication** | **Comments** |
| --- | --- |
| Decrease in kidney function | Increased risk of hyperkalemia, hyperphosphatemia, metabolic acidosis, hypermagnesemia; sodium may increase or decrease depending on the water and sodium intake/losses |
| Improvement in kidney function | Increased risk of hypokalemia, hypophosphatemia or hypomagnesemia if intake restricted or non-renal losses; sodium may also decrease or increase; daily monitoring often adequate since changes usually occur gradually |
| Decrease in urine output | Beyond risks of decreased kidney function, risk of hyponatremia increases given inability to excrete water |
| Increase in urine output | Beyond risks of improved kidney function, risk of hyponatremia or hypernatremia increases given urinary losses of sodium and water |
| Intermittent dialysis | Increased risk of hypophosphatemia, hypokalemia, and hypomagnesemia, especially if intake restricted |
| CRKT | Increased risk of hypophosphatemia, hypokalemia, metabolic alkalosis and hypomagnesemia, but risk highly dependent on composition of dialysis/replacement fluid. Risk may be higher with CKRT than intermittent dialysis but depends on composition of dialysis/replacement fluid. |
| Citrate anticoagulation for CKRT | Increased risk of citrate lock (elevated total calcium with normal ionized calcium) |
| Discontinuation of dialysis | Increased risk of hyperkalemia, hyperphosphatemia, metabolic acidosis, hypermagnesemia; sodium may increase or decrease depending on the water and sodium intake/losses. Risk may be higher with CKRT than intermittent dialysis but depends on composition of dialysis/replacement fluid. Anticipation of these changes and adjustments of electrolyte delivery prior to discontinuation is ideal |
| CKRT, continuous kidney replacement therapy | |

**Supplementary Table 5. Medication-based causes of electrolytes disturbances**

| **Electrolyte Disturbance** | **Medication** |
| --- | --- |
| Hyponatremia | - Thiazide diuretics - Loop diuretics - ACE inhibitors - Sulfamethoxazole/trimethoprim - NSAIDs - Tricyclic antidepressants - Selective serotonin reuptake inhibitors - Monoamine oxidase inhibitors - Carbamazepine, oxcarbazepine, sodium valproate - Lamotrigine - Vinca alkaloids - Platinum compounds |
| Hypernatremia | - Hypertonic saline - Amphotericin B - Foscarnet - Lithium, phenytoin - Corticosteroids, mineralocorticoids - Osmotic cathartic agents |
| Hypokalemia | - Insulin - Aminophylline, theophylline - Caffeine - Terbutaline - Albuterol - Loop diuretics - Thiazide diuretics - Aminoglycosides - Amphotericin B - Sodium polystyrene sulfonate - Corticosteroids, mineralocorticoids |
| Hyperkalemia | - Penicillin - Spironolactone, eplerenone - Trimethoprim - Beta blockers - Digoxin - Ace inhibitors/Angiotensin receptor blockers - Cyclosporine, tacrolimus - NSAIDs - Azole antifungals |
| Hypophosphatemia | - Aluminum or magnesium containing antacids - Sucralfate - Phosphate binders - Albuterol - Insulin - Sodium bicarbonate |
| Hyperphosphatemia | - Phosphate-containing enema or laxative |
| Hypocalcemia | - Bisphosphonates - Calcitonin - Amphotericin B - Foscarnet - Citrate - Phenytoin, phenobarbital - Rifampicin, isoniazid - Estrogen - Loop diuretics - Aminoglycosides |
| Hypercalcemia | - Vitamin D - Estrogen - Tamoxifen - Loop diuretics |
| Hypomagnesemia | - Aminoglycosides - Amphotericin B - Cisplatin, methotrexate - Cyclosporine - Thiazide and loop diuretics - Foscarnet |
| Hypermagnesemia | - Insulin - Magnesium-containing enemas/laxatives/antacids |
| ACE, angiotensin-converting enzyme; NSAID, non-steroidal anti-inflammatory drug | |
